# Rational Design of Magnetic Nanoparticles as T_1_–T_2_ Dual-Mode MRI Contrast Agents

**DOI:** 10.3390/molecules29061352

**Published:** 2024-03-18

**Authors:** Carlos F. G. C. Geraldes

**Affiliations:** 1 Department of Life Sciences and Coimbra Chemistry Center (CQC-IMS), Faculty of Science and Technology, University of Coimbra, 3004-531 Coimbra, Portugal; geraldes@ci.uc.pt; Tel.: +351-967661211; 2 CIBIT—Coimbra Institute for Biomedical Imaging and Translational Research, University of Coimbra, 3004-531 Coimbra, Portugal

**Keywords:** magnetic nanoparticle, magnetic resonance imaging, positive contrast, negative contrast, bimodal contrast agent

## Abstract

Magnetic nanoparticles (MNPs), either paramagnetic or superparamagnetic depending on their composition and size, have been thoroughly studied as magnetic resonance imaging (MRI) contrast agents using in vitro and in vivo biomedical preclinical studies, while some are clinically used. Their magnetic properties responsible in some cases for high magnetization values, together with large surface area-to-volume ratios and the possibility of surface functionalization, have been used in MRI-based diagnostic and theranostics applications. MNPs are usually used as positive (T_1_) or negative (T_2_) MRI contrast agents, causing brightening or darkening of selected regions in MRI images, respectively. This review focusses on recent developments and optimization of MNPs containing Gd, Mn, Fe and other lanthanide ions which may function as dual-mode T_1_–T_2_ MRI contrast agents (DMCAs). They induce positive or negative contrast in the same MRI scanner upon changing its operational mode between T_1_-weighted and T_2_-weighted pulse sequences. The type of contrast they induce depends critically on their r_2_/r_1_ relaxivity ratio, which for DMCAs should be in the 2–10 range of values. After briefly discussing the basic principles of paramagnetic relaxation in MNPs, in this review, the basic strategies for the rational design of DMCAs are presented and typical examples are discussed, including in vivo preclinical applications: (1) the use of NPs with a single type of contrast material, Gd- or Mn-based NPs or superparamagnetic NPs with appropriate size and magnetization to provide T_2_ and T_1_ contrast; and (2) inclusion of both types of T_1_ and T_2_ contrast materials in the same nanoplatform by changing their relative positions.

## 1. Introduction

Magnetic resonance imaging (MRI) is one of the most prominent clinical imaging modalities resulting from its many favorable characteristics. These include non-invasiveness, use of low-energy radiofrequency radiation, in contrast to invasive X-ray computed tomography (CT), positron-emission tomography (PET) and single-photon emission computed tomography (SPECT), which use damaging high-energy ionizing radiation, providing tomographic images with large penetration depth of any area of the body, in contrast to optical imaging (OI), outstanding spatial resolution (50–100 μm) and remarkable soft tissue contrast (see Table 1 for a comparison of some properties of imaging modalities for clinical applications). The contrast in the MRI images is generated by differences in intensities of ^1^H NMR resonances, most frequently of water protons, with an important contribution of lipids in some cases. These intensities are governed by several parameters, including the local tissue ^1^H concentrations, diffusion and flow of water molecules, and most importantly by their intrinsic differences in proton spin–lattice or longitudinal relaxation times (T_1_) and spin–spin or transverse relaxation times (T_2_) [1,2]. However, the sensitivity of MRI is relatively low, due to the small population difference between the two proton spin energy states in the presence of a magnetic field, making it difficult to detect small lesions and limiting the time resolution of the technique. In recent decades, there has been a trend toward clinical MRI equipment operating at higher magnetic field strengths (B_0_), which improved its contrast-to-noise ratios and spatial resolution. Presently, medical diagnostic MRI is typically performed at B_0_ = 0.5–3 T, and research equipment can operate at B_0_ values up to 11 T.

MRI contrast agents (CAs) are often employed to enhance the contrast between normal and disease tissues by significantly decreasing their T_1_ and T_2_ values [3,4,5,6,7]. This acceleration of the spin relaxation of water protons, in the region where they accumulate relative to surrounding tissues, results from oscillating magnetic fields produced by metal ions present in the CAs, allowing sensitive MRI detection of that region. Their efficacy is measured by their relaxivity, r_1_ or r_2_, defined as the paramagnetic enhancement of the water proton relaxation rates, normalized to a 1 mM metal ion concentration [3,4,5].

**Table 1:** Comparison of some typical properties of imaging modalities for clinical applications [8,9].

Technique	Resolution	Penetration Depth	Sensitivity
MRI	50–100 μm	No limit	10^−4^–10^−5^ mM
PET	1–2 mm	No limit	pM
SPECT	1–2 mm	No limit	nM
OI	2–5 mm	<2 cm	<<nM
CT	50–200 μm	No limit	0.1 mM ^a^

^a^ For an iodine-containing CA.

A class of MRI CAs contains strongly paramagnetic metal ions, such as Gd^3+^, Mn^2+^ and Fe^3+^, encapsulated by a strongly binding chelating ligands, or in the form of paramagnetic nanoparticles (MNPs). These CAs increase the longitudinal relaxation rates of water protons significantly more than their transverse relaxation rates, giving rise to bright spots in T_1_-weighted (T_1w_) MRI images and are called positive or T_1_ CAs [3,4,5,6,7,10]. Another class of CAs, containing paramagnetic metal ions, such as Tb^3+^, Dy^3+^ and Ho^3+^, in the form of paramagnetic chelates or NPs, or as superparamagnetic NPs, such as iron-oxide NPs (SPIONs), induce protons in their vicinity to preferably undergo spin–spin relaxation, originating negative (or dark) contrast in T_2_-weighted MRI images and are called negative or T_2_ CAs. In addition to r_1_ and r_2_, the r_2_/r_1_ ratio (always ≥1, as T_2_ ≤ T_1_) represents another important factor in identifying the class of MRI CAs. This ratio should be close to one for a T_1_ CA (positive contrast), while it is large for a T_2_ CA [10,11,12,13].

Nowadays, approximately 40% of the MRI exams are carried out after administration of a CA. The materials used as CAs for clinical studies must meet the following requirements: (1) high relaxivity; (2) high water solubility or colloidal stability; (3) low osmolality to avoid pain from osmotic shock and other adverse effects upon injection; (4) low toxicity, which requires both high thermodynamic and kinetic stability (no transmetallation by endogenous metal ions such as Zn^2+^) in the case of metal chelates, and no leaching of toxic free metal ions in vivo from NPs; (5) rapid excretion, ideally not much longer than the MRI exam time; (6) a biodistribution with high specificity for the area of interest.

Various MRI contrast agents have been investigated and developed to achieve this goal. Gd-based CAs (GBCAs) include macrocyclic chelates of open-chain DTPA-type (DTPA = diethylenetriaminepentaacetic acid) or macrocyclic DOTA-type (DOTA = 2,2′,2″,2′″-(1,4,7,10-tetraazacyclododecane-1,4,7,10-tetrayl) tetraacetic acid) derivatives, such as Magnevist^®^ (Bayer Schering Pharma AG, Berlin, Germany), Dotarem^®^ (Guerbet, Paris, France), Omniscan^®^ (GE Healthcare, Chicago, IL, USA) and ProHance^®^ (Bracco, Milan, Italy) [7], which are clinically used as T_1_ MRI CAs. These molecular agents have low r_1_ and r_2_ values and short blood circulation times due to their efficient renal excretion, and thus large amounts of injection doses are needed (typically 0.1 mmol/kg body weight) to achieve detectable contrast levels. This can increase the risk of toxicity due to potential release of free Gd^3+^ ions in the body [14]. This has occurred in some cases to patients with chronic kidney disease, which developed nephrogenic systemic fibrosis (NSF), characterized by skin thickening and hyperpigmentation and extracutaneous fibrosis [15,16,17]. In addition, recent studies indicated that clinically developed GBCAs could be deposited in the brain after repeated use, and could cause neurotoxicity, although this has not been proven [18,19]. In both cases, tissue deposits of linear GBCAs are much higher than those of macrocyclic GBCAs.

Biocompatible dextran-coated superparamagnetic iron oxide nanoparticles (SPIONs) Feridex^®^ (Bayer HealthCare Pharmaceuticals, Wayne, NJ, USA), Sinerem^®^(Guerbet, Paris, France), and Resovist^®^(Bayer Schering Pharma AG, Berlin, Germany), have been approved by the Food and Drug Administration (FDA), USA, for clinical trials in liver (Feridex^®^ and Resovist^®^) and lymph nodes (Sinerem^®^) MRI, as well as Lumirem^®^ (Guerbet, Paris, France) for gastrointestinal imaging. However, Feridex^®^ and Sinerem^®^ were withdrawn from the market, mostly for commercial reasons. Nowadays, only Resovist^®^ is used for liver imaging in some countries [7,20].

Magnetic NP (MNP)-based MRI CAs have many advantages relative to metal chelates, including larger magnetic moments and longer blood circulation times, which cause higher image contrast. They can also be used as multifunctional nanoplatforms for multimodal imaging, therapy and drug delivery (theranostics) after surface functionalization. [21]. MNPs are composed of two parts: a magnetic core that enhances MRI and a surface-coating ligand layer responsible for colloidal stability and minimizes toxicity. This review focusses on recent developments and optimization and in vivo applications of MNPs containing Gd, Mn, Fe and other lanthanide ions which may function as dual-mode T_1_–T_2_ MRI contrast agents (DMCAs). As the kind of contrast provided by MNPs depends fundamentally on their r_2_/r_1_ ratios, the description of the principles for rational design of DMCAs starts by a brief summary of the basic theory of paramagnetic relaxation in MNPs. Then, as their r_2_/r_1_ ratios are critically dependent on the MNPs composition, size and surface-coating, the different ways in which these properties can be modulated to design T_1_ or T_2_ single-mode MRI CAs or T_1_–T_2_ DMCAs are illustrated using selected examples. As the MNPs composition is a very important factor, a particular attention is given to systems using a single type of contrast material (e.g., Gd- or Mn-based T_1_ contrast materials or superparamagnetic T_2_ contrast materials) or both types of contrast materials in the same nanoplatform.

## 2. Basic Principles of Paramagnetic Relaxation in Small Complexes and Nanoparticles

The observed longitudinal proton relaxation rate ($R_{1}^{obs}$) of a small paramagnetic complex in aqueous solution is the sum of a paramagnetic and a diamagnetic term, where the first is proportional to the concentration of the paramagnetic ion and the second is the contribution of the water solvent:

(1) $$R_{1}^{obs}=R_{1}^{p}+R_{1}^{d}=r_{1}[Mn]+R_{1}^{d}$$

The observed relaxivity (in mM^−1^ s^−1^ units) is the sum of the inner (r_1is_) and outer sphere (r_1os_) terms:

(2) $$\;r_{1}=r_{1}^{is}+r_{1}^{os}$$

The same equations apply to R_2_ and r_2_. These contributions are evaluated using the dominant dipole–dipole relaxation mechanism, which is modeled by the Solomon–Bloembergen–Morgan (SBM) theory for r_1is_, and the Freed theory for r_1os_ [3,4,5,6,22]. For r_1_, the main parameters determining the IS contribution are the number of water molecules in the first coordination sphere of the metal ion (q), and the three processes responsible for the time fluctuation of the nucleus–electron interactions, which are the inner-sphere water residence time (τ_M_ = k_ex_^−1^, where k_ex_ is the water exchange rate), the molecular reorientational correlation time (τ_R_) and the electron spin relaxation time (T_1e_) of the metal ion, which is frequency dependent. The OS contribution is determined by the diffusion correlation time (τ_D_) and the distance of closest approach (a) of the water molecules freely diffusing near the complex.

The r_1_ and r_2_ values of paramagnetic NPs also have in principle IS and OS contributions. The IS contribution results from the exchange of the water protons directly coordinated to the metal ions at the NPs surface with bulk water, as those located below the surface have a negligible effect. The IS r_1_ depends on the hydration number of the surface ions and the NP’s surface-to-volume ratio [23]. In the case of Gd_3+_ ions, they affect the bound water proton relaxation mainly through the dipolar mechanism, which can be modeled by the SBM equations. When other paramagnetic Ln^3+^ ions with high magnetic moments and very short T_1e_ values (e.g., Tb^3+^, Dy^3+^, Ho^3+^) (Table 2) are present, a Curie term is also present [24,25,26].

However, for surface-coated NPs, the OS contribution dominates the T_1_ and T_2_ relaxation, as the water protons are indirectly in contact with the paramagnetic metal ions in a NP due to the surface coating. It results from the diffusion of water molecules in the fluctuating magnetic field inhomogeneities created in their vicinity by the magnetized NPs, and does not contain the Curie contribution. The r_1_ value is approximately proportional to the square of the spin magnetic moment (μ_s_^2^ = S(S + 1)ħ^2^ for transition metal ions and μ_s_^2^ = 4S(S + 1) + L(L + 1)ħ^2^ for Ln^3+^ ions, where S is the spin quantum number and L is the orbital quantum number) multiplied by the number (N) of Ln^3+^ ions in a NP which can interact with a water proton, as given by Equation (3):

r_1_ ∝ Nμ_s_^2^ (3)

Transverse relaxation of water protons is induced by fluctuations of local magnetic fields generated by the NPs. Thus, r_2_ is proportional to the square of the total magnetic moment (μ) of the NP, as given by Equation (4):

r_2_ ∝ μ^2^ (4)

In the case of superparamagnetic NPs, the OS contribution is also dominant. However, for a colloidal dispersion in the presence of a magnetic field, the return of their magnetization to equilibrium is determined by two different processes [11]: (a) the Néel relaxation, describing the return of the magnetization of each of the NPs to equilibrium after a perturbation that tilts that magnetization away from the direction of its easy axis; its relaxation time (τ_N_) defines the fluctuations that arise from jumps of the magnetization between different easy directions; (b) Brownian relaxation, defined by the relaxation time τ_B_, which characterizes the viscous rotation of the particle. The global magnetic relaxation rate of the colloid is the sum of the Néel (τ_N_^−1^) and Brownian (τ_B_^−1^) relaxation rates, τ^−1^ = τ_N_^−1^ + τ_B_^−1^, where τ is the global magnetic relaxation time. In these systems, r_1_ and r_2_ can be described by Freed’s model for paramagnetic systems, using the diffusion correlation time (τ_D_) and considering the electron spin longitudinal relaxation time τ_S1_ as equal to the Néel relaxation time τ_N_.

The magnetization of the superparamagnetic iron oxide NPs reaches its saturation value M_S_ at B_0_ ≤ 0.5 T, which is at the lower limit for commonly used clinical MRI scanners. Therefore, their R_2_ is in practice usually independent of B_0_. The r_1_ value is governed by the volume fraction of the superparamagnetic particles (υ), the diffusion correlation time (τ_D_ = d^2^/4D, where d is the diameter of the particle and D is the diffusion coefficient), and the magnetization of the NP (M_S_) at the B_0_ value of the clinical MRI scanner. A discussion of the transverse relaxivity of spherical superparamagnetic NPs can be found in the literature [11,27,28,29].

Superparamagnetic NPs are the T_2_ CAs most commonly used in MRI, usually with a very high r_2_ (typically 60–400 s^−1^mM^−1^), in particular the SPIONs. This can hamper the interpretation of T_2w_ images due to the difficulty in distinguishing the CA-induced darkening from partial-volume artifacts, motion artifacts, and tissue inhomogeneities [30].

## 3. Magnetic Nanoparticles as T_1_–T_2_ Dual-Mode MRI Contrast Agents

### 3.1. NPs for T_1_ or T_2_ Single-Mode MRI Contrast

The type of contrast provided by MNPs as MRI CAs depends on their r_1_ and r_2_ values, as well as their r_2_/r_1_ ratios, which are dependent on their composition, size and surface-coating [31]. The ideal T_1_ MRI CA should have high r_1_ values and r_2_/r_1_ ratios close to 1.0. The most important contribution to the r_1_ values comes from the metal ions present on the MNP surface, which interact directly with nearby water proton spins by an IS mechanism. The most efficient paramagnetic ions are Gd^3+^ (S = 7/2), Mn^2+^ (S = 5/2), and Fe^3+^ (S = 5/2) (S is the total spin quantum number) due to their high number of unpaired electrons, high magnetic moments and long T_1e_ values (Table 2) [3,4,5,6,22].

Several inorganic Gd^3+^-containing NPs with the largest possible Gd^3+^ densities [32], such as Gd_2_O_3_ [33,34], Gd_2_O_2_S [35], Gd-carbonates [36], GdF_3_ [37,38], and GdPO_4_ [39,40], have been proposed as potential T_1_ MRI CAs. Small (<10 nm diameter) Gd_2_O_3_ NPs have been the most intensively investigated, stabilized by coating with D-glucuronic acid [41], polyvinylpyrrolidone (PVP) [42], or polyethylene glycol (PEG) [43]. Paramagnetic NPs incorporating Gd^3+^ into the particle core or shell in a core/shell construct with large magnetization values have been proposed as T_1_ MRI CAs. An example of this approach is NaYF_4_:Yb, Er@NaGdF_4_ core/shell NPs (20−40 nm diameter), with Gd^3+^ positioned only on the outer shell, where it can interact directly with water protons and promote T_1w_ image contrast [44].

Many small (<10 nm diameter) Mn-containing NPs, including MnO (Mn^2+^), Mn_2_O_3_ (Mn^3+^) and MnO_2_ (Mn^4+^), have also been studied as T_1_ MRI CAs [45,46,47,48]. Their r_1_ values depend strongly on their geometry and morphology, as their interfaces with water critically influence their contrast effects. Some typical examples are zwitterionic dopamine sulfonate (ZDS)-coated ultrasmall MnO NPs (USMnO@ZDS) [49], PEG-AS1411 aptamer-coated MnO (AS1411-PEG-MnO) NPs [50] and ligand-free Mn_3_O_4_ NPs [51]. Non-oxide Mn^2+^-containing materials, such as other inorganic (i.e., KMnF_3_, MnWO_4_), Mn^2+^-chelate-based, hybrid organic/inorganic and Mn^2+^-based layered double hydroxide (Mn-LDH) NPs have also been exploited as T_1_ MRI probes.

The use of high spin Fe^3+^ ions as T_1_ MRI probes within nanostructures for in vitro studies or preclinical investigations has been recently reviewed [52], including examples such as amphiphilic polymer-based NPs like Fe^3+^-chelated poly(lactic-co-glycolic) acid (PLGA) NPs, NPs containing polyphenolic Fe^3+^-binding units such as Fe^3+^-loaded synthetic melanin nanoparticles (SMNPs) and bimetallic (Gd, Fe)-phenolate coordination polymer (CN) NPs. In several of these systems, although the Fe^3+^ ion is not hydrated (q = 0) and therefore the IS (q ≥ 1) contribution to relaxivity is not present, the reported r_1_ values are higher than those expected for the OS (q = 0) contribution alone, which suggests the presence of a significant contribution from the water molecules of the second hydration sphere (SS).

The ideal T_2_ MRI CAs should have high r_2_ values and high (≥10) r_2_/r_1_ ratios to induce predominantly T_2_ proton spin relaxation, leading to a decrease in signal intensity in T_2w_ or T_2w_* MRI images [31]. The systems that fulfil these conditions are SPIONs and paramagnetic lanthanide (Ln^3+^)-based NPs (Ln = Tb, Dy and Ho), specially at high magnetic fields (B_0_).

As discussed in Section 2, SPIONs are the T_2_ CAs most commonly used in MRI, having a very high r_2_ (usually 60–400 s^−1^mM^−1^). Examples are Resovist^®^ (a carboxydextran-coated SPION), octapod NPs with higher r_2_ values than spherical NPs, highlighting the importance of size and morphology optimization to obtain NPs with high r_2_ [53], ultrasmall SPIONs (USPIONs) with a scaffold of bovine serum albumin (BSA) [54], SPIONs conjugated with PEG of molecular weight (MW) in the 600–8000 amu range [55] and ferrimagnetic iron oxide nanocubes (FIONs) encapsulated in PEG phospholipids to become water-dispersible (WFIONs) [56]. The WFIONs have an extremely high r_2_ value of 761 mM^−1^.s^−1^ at 3 T.

Paramagnetic NPs containing Tb^3+^, Dy^3+^ or Ho^3+^ have high r_2_ values due to their high magnetic moments and very short (<1 ps) electronic relaxation times (Table 2) [24]. Therefore, they can be alternatives to SPIONs as efficient T_2_ and T_2_* CAs [13,57,58,59,60], in particular at high MRI fields (B_0_ > 3 T). This is because r_2_ generally increases with B_0_, but the contribution from the Curie spin relaxation mechanism increases with B_0_^2^. The maximum r_2_ values are obtained at slower inner sphere water exchange (τ_M_ = 0.1–10 μs) than for r_1_ (τ_M_ = 1–100 ns) [61]. Also, in contrast to iron oxide particles, Ln^3+^-containing NPs show no saturation of the magnetization, even at magnetic field strengths as high as 30 T [62].

The highest payload of Ln^3+^ ions per particle at a particular site can be delivered by inorganic Ln^3+^-containing NPs, including Ln_2_O_3_, LnF_3_, and NaLnF_4_. NPs with a diameter of 50–100 nm contain approximately 106 Ln^3+^ ions each and their magnetic properties make them good candidates for T_2w_ and T_2w_* MRI CAs [63]. Dy^3+^-based nanomaterials are the most studied, as they show the largest T_2_ relaxation effects [23]. Moreover, the T_2_ effects for Dy^3+^-based nanomaterials, such as β-NaDyF_4_, are two orders of magnitude higher than for clinically approved iron oxide nanomaterials under the increasingly used very high field magnets (7 T and higher). This arises due to the Curie spin relaxation mechanism of the Dy^3+^-based systems [64]. However, coating of the particles is necessary to avoid leaching of free Ln^3+^.

### 3.2. NPs as T_1_–T_2_ DMCAs

While conventional MRI CAs respond only in a single imaging mode, either T_1_ or T_2_, NPs with multimodal capabilities provide complementary diagnostic information. However, when hybrid imaging systems, such as PET/CT or PET/MRI scanners are not available, a combination of two different imaging devices must be used separately, which is an inconvenient, time-consuming and expensive procedure [65,66,67,68,69]. The development of MRI T_1_–T_2_ dual-mode CAs in a single nanoplatform is an attractive solution to overcome ambiguities in conventional MRI diagnostics, especially when the biological targets are small, as well as the image matching difficulties caused by relocating the imaging object, and by the discrepancies resulting from different depth penetrations and spatial/time resolutions of multiple imaging strategies [69]. In fact, T_1_–T_2_ dual-modal MRI images can be easily acquired by changing the parameters of the pulse sequences in the operational mode of the same MRI scanner.

Dual-modal T_1_–T_2_ MRI CAs (DMCAs) should have both high r_1_ and r_2_ values, with r_2_/r_1_ ratios (~2–10) between those of ideal T_1_ and T_2_ MRI CAs. If Gd-based NPs are to be used as DMCAs, their r_2_/r_1_ ratios should be increased from their usual values of ~1, while the use of SPION-based DMCAs requires a decrease in their r_2_/r_1_ ratios from their usual very high values. Mn-based NPs are useful as DMCAs because of their suitable r_2_/r_1_ ratios.

The approaches proposed to design DMCAs comprise: (1) use of NPs with a single type of contrast material, Gd- or Mn-based NPs or superparamagnetic NPs with appropriate size and magnetization to provide T_2_ and T_1_ contrast; or (2) include both T_1_ and T_2_ contrast materials in the same NP. Both have advantages and disadvantages [31,69,70,71,72].

#### 3.2.1. DMCAs Based on a Single Type of Contrast Material

1. NPs based on a typical T_1_ agent

This strategy is based on the clustering of a T_1_ contrast material within non-magnetic porous matrices to obtain enhanced r_1_ and specially r_2_ values. The T_1_ contrast material includes Gd-based complexes (e.g., Gd (DOTA)) or NPs (e.g., Gd_2_O_3_) [73,74,75,76], Mn-based NPs (e.g., MnO, MnO_x_) or Mn^2+^ ions [77,78,79], mixed Gd/Dy NPs [80,81], and Fe^3+^ cations [82,83,84] entrapped in porous matrices, such as polymers (e.g., poly(amidoamine) (PAMAM) dendrimers, poly-(α,β)-DL-aspartic acid (PASA), mesoporous poly dopamine (MPDA)), porous silica, proteins (e.g., bovine serum albumin (BSA), hydrogels (e.g., hyaluronic acid (HA)/CH (chitosan)) or organic frameworks (e.g., NCP and CP_3_ coordination polymers)) (Table 3). This entrapment of metal chelates and NPs by serum proteins is likely to occur by opsonization [76]. Some DMCAs have also theranostic properties, by including drugs such as doxorubicin (DOX) in the matrices [78,79,83,84]. The ideal range of r_2_/r_1_ ratio can be approached by increasing r_2_ of water protons through restricting water diffusion around the complex through clustering [73]. Another possibility is to decrease r_1_ by restraining the metal ion coordination and chemical exchange of surrounding water molecules through geometrical confinement.

**Figure 1:**
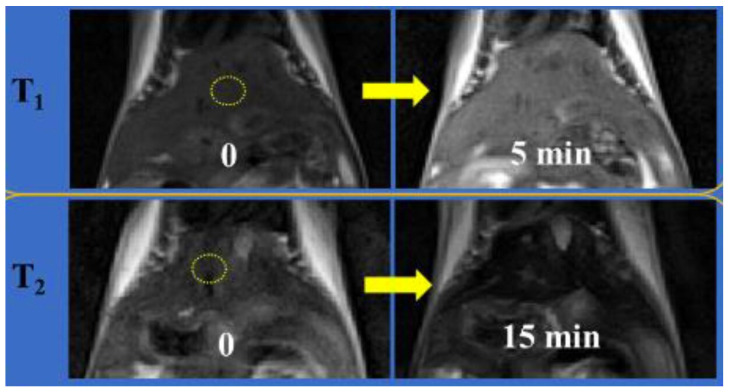
T_1_ and T_2_ MR images of mice liver before (labeled as “0”) and 5 and 15 min after intravenous injection of an aqueous solution of PASA-coated Gd_2_O_3_ nanoparticles into mice tails, respectively (N_mice_ = 2 for each modal imaging). The echo time = 10 (or 37) ms, repetition time = 385 (or 1620) ms, pixel bandwidth = 299 (or 197) Hz, number of acquisitions = 8 (or 4), echo train length = 3 (or 13), flip angle = 120° (or 120°), slice thickness = 1.0 (or 1.0) mm, and slice gap = 1.1 (or 1.1) mm were used for T_1_ (or T_2_) MR image measurements. Reproduced from [74].

A few of the systems summarized in Table 3 are discussed here in more detail. Marasini et al. reported a Gd-based T_1_–T_2_ DMCA consisting of Gd_2_O_3_ NPs (2 nm diameter) coated with the hydrophilic polymer PASA using the one-pot polyol method. The NPs had r_1_ = 19.1 mM^−1^.s^−1^ and r_2_ = 53.7 mM^−1^.s^−1^ (r_2_/r_1_ = 2.8) at 3 T. After i.v. injection of Gd_2_O_3_@PASA NPs into the mice tail vain, T_1_ and T_2_ contrasts were observed in the T_1w_ and T_2w_ MRI images of the mouse livers at 3 T, respectively (Figure 1) [74].

**Figure 2:**
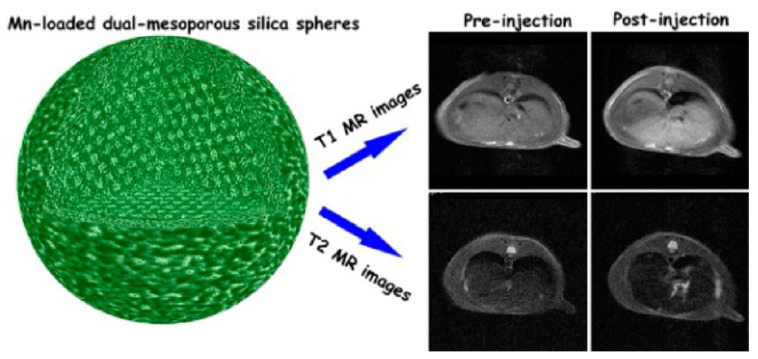
(**Left**): a model structure of Mn-DMSS NPs; (**Right**): Simultaneous T_1_ and T_2_ imaging of a SD rat liver on a 3.0 T MRI scanner: In vivo T_1_-weighted (**top**) and T_2_-weighted (**bottom**) MR images. Reproduced with permission from [77]. Copyright from the American Chemical Society.

A Mn-based T_1_–T_2_ DMCA was reported consisting of dual mesoporous silica spheres loaded with a MnO nanocluster (diameter < 2 nm) (Mn-DMSS) with a hydrodynamic diameter in the 100–200 nm range (Figure 2), with r_1_ = 10.1 mM^−1^.s^−1^ and r_2_ = 169.7 mM^−1^.s^−1^ (r_2_/r_1_ = 16.8) at 3 T. In vivo MRI experiments on Sprague Dawley (SD) rats at 3 T showed a 29% signal enhancement in the liver in T_1w_ images and a 28% signal decrease in T_2w_ images upon injection of Mn-DMSS NPs (Figure 2) [77].

A Gd/Dy-based novel PEGylated Dy-doped NaGdF_4_ nanoprobe (NaGdF_4_:Dy @DSPE-PEG_2000_) was developed as a T_1_/T_2_-weighted MRI/CT imaging nanoplatform, with r_1_ = 5.17 mM^−1^.s^−1^ and r_2_ = 10.64 mM^−1^.s^−1^ (r_2_/r_1_ = 2.4) at 9.4 T and strong X-ray attenuation properties (44.70 HU L g^−1^) in vitro. T_1w_/T_2w_ MRI/CT imaging in vivo of mice injected with the nanoprobe led to significant contrast enhancement of liver, spleen and kidneys at 24 h post injection. This multifunctional nanoprobe had low cytotoxicity and was completely excreted from the injected mice [81].

Finally, a Fe-based dual-mode T_1_–T_2_ MRI DMCA was reported consisting of a one-pot synthesized nanostructured coordination polymer containing Fe^3+^ (Fe-NCP) functionalized with BSA. These NPs, with a hydrodynamic diameter of 97 nm and a ζ potential of −31.2 mV, had high colloidal stability, low cytotoxicity and good relaxivities, r_1_ = 5.3 mM^−1^.s^−1^ and r_2_ = 10.9 mM^−1^.s^−1^ (r_2_/r_1_ = 2.1) at 7.0 T. Their T_1_–T_2_ MRI contrast was verified in in vitro phantoms, ex vivo C57BL/6J mice and in vivo GL261 glioblastoma tumor-bearing mice. The vivo MRI of Fe−NCPs showed high T_1_ and T_2_ contrast in the tumor in a very short period of time and were safe for the mouse. Their large long-term uptake in the spleen was related to their rapid clearance by the mononuclear phagocytic system (MPS) due to the NPs size being bigger than 40 nm [83].

Table 3 shows that not all the systems described reach the low r_2_/r_1_ values recommended for efficient DMCAs, illustrated by the second and fourth examples discussed above [77,83].

2. NPs based on a typical T_2_ agent: USPIONs and FeO_x_

This strategy is based on using superparamagnetic NPs with appropriate size and magnetization to provide T_2_ and T_1_ contrast simultaneously. Although SPIONs with a core diameter less than 10 nm can produce positive contrast in T_1w_ images at low concentrations, their high T_2_ effects (high r_2_/r_1_) resulting from their high magnetic moment limit their application as T_1_–T_2_ DMCAs. However, the magnetic moment of Fe_3_O_4_ NPs is strongly dependent on their size and decreases rapidly as their size decreases due to the reduction in the volume magnetic anisotropy and spin disorder (canting) at their surface, which suppresses the T_2_ effect and therefore maximizes the T_1_ contrast effect, controlling their relaxivities [11]. Therefore, the appropriate ultra-small-sized Fe_3_O_4_ NPs (USPIONs) are potential candidates for T_1_–T_2_ DMCAs.

Some examples of such systems are summarized in Table 4, where the USPION core was coated with hydrophilic polymers, e.g., poly(methacrylic acid) (PMAA)-polytrimethylene terephthalate (PMAA-PTTM) [85], poly(acrylic acid) (PAA) [86,87] and PEG [88], or silica [89], to prevent the aggregation of the NPs and to ensure a small particle size. The core was usually spherical, but in some cases nanoplates [89] or nanocubes [90,91] were used. Some of the systems summarized in Table 4 are now discussed in more detail.

**Figure 3:**
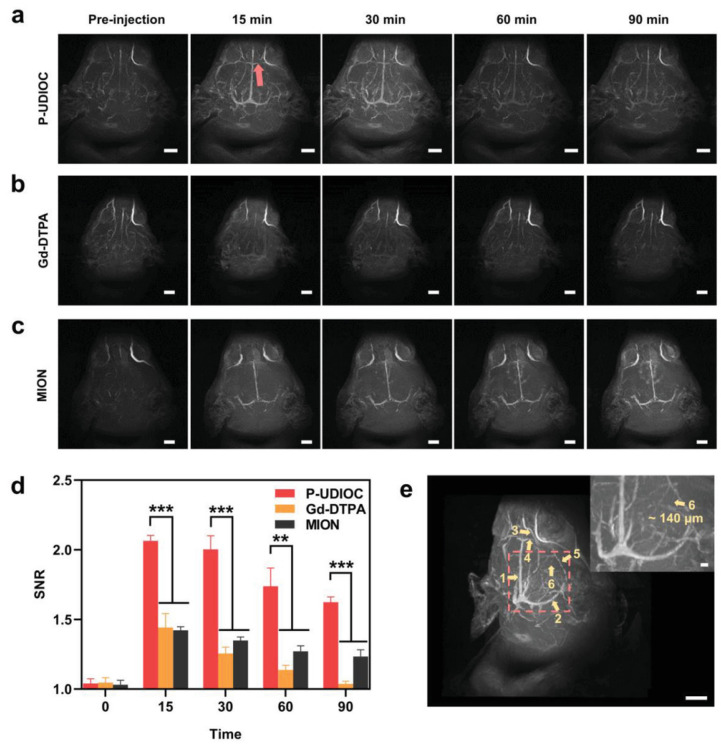
Vascular imaging performance of P-UDIOC-enhanced UHF MRI. (**a**) P-UDIOC, (**b**) Gd-DTPA and (**c**) MION enhanced MRA maximum intensity projection images of the rat brain (scale bar = 5 mm). (**d**) Quantitative analysis of vascular imaging performance by calculating SNR values of inferior cerebral vein indicated by the red arrow in a before and after i.v. injection of P-UDIOC, Gd-DTPA, and MION (*n* = 3). Statistical analysis was performed using a Student’s *t*-test, with *** indicating *p* < 0.001, ** indicating *p* < 0.01. (**e**) 3D volume image of the rat brain at 15 min post-injection of P-UDIOC, showing high spatial resolution of the vasculature (scale bar = 5 mm). The yellow arrows and labeled numbers in the image show the vascular details including 1, superior sagittal sinus; 2, transverse sinus; 3, anterior cerebral artery; 4, inferior cerebral vein; 5, basal vein; 6, middle meningeal artery. The inset image in (**e**) is an enlargement of the region indicated by the red dashed square (scale bar = 500 μm). Reproduced from [88].

Li et al. reported monodispersed water-soluble and biocompatible USPIONs (3.3 nm average diameter) grafted with thiol functionalized (PMAA)-(PTTM) using a high-temperature co precipitation method. These NPs, with r_1_ = 8.3 and r_2_ = 35.1 s^−1^.mM^−1^ (r_2_/r_1_ = 4.2) at 4.7 T, showed in vitro and in vivo potential as T_1_–T_2_ DMCAs upon i.v. injection in mice, as positive and negative contrasts were observed in the T_1w_ and T_2w_ MRI images of liver and kidneys, respectively [85]. Miao et al. developed PAA-coated USPIONs (5.1 nm core diameter and 41.35 nm average hydrodynamic size) with r_1_ = 10.52 and r_2_ = 38.97 s^−1^.mM^−1^ (r_2_/r_1_ = 3.70) at 1.41 T. The NPs had in vitro and in vivo potential as T_1_–T_2_ DMCAs, as illustrated by T_1w_ and T_2w_ MRI images at 3 T, with positive contrast observed in the rabbit vasculature, while the rabbit popliteal lymph node exhibited negative contrast [87]. Wang et al. reported USPION-PEG (P-UDIOC) NPs with extremely small core size (2.3 nm) and a compact hydrophilic PEG surface and with r_1_ = 1.37 and r_2_ = 7.53 s^−1^.mM^−1^ (r_2_/r_1_ = 5.5) at the ultrahigh field (UHF) of 7.0 T. These UHF-tailored T_1_–T_2_ DMCAs showed dual enhanced T_1_–T_2_ contrast at 7 T and enabled a clear visualization of microvasculature as small as ≈140 μm in diameter under UHF MRI (Figure 3), extending the detection limit of 7 T MRI angiography (MRA) [88].

An example of a system with a non-spherical core consisting of Fe_3_O_4_ crystal nanoplates was reported by Zhou et. al. [89] These superparamagnetic magnetite nanoplates had (111) exposed facets which much contributed to their T_1_ and T_2_ contrast effects. The main contribution to r_1_ of the magnetic nanoplates is the chemical exchange with bulk water (given by the exchange lifetime, τ_M_) of the water molecules bound at the inner-sphere of the Fe^3+^ ions on the highly exposed particle surface iron-rich Fe_3_O_4_ (111) surfaces, according to the dominant IS model [11]. The r_2_ values are dominated by the OS mechanism, which accounts for the effect of fluctuations of the local magnetic field inhomogeneities induced by the tumbling NPs on the protons of outer-sphere water molecules diffusing nearby. According to Freed’s theory, the r_2_ value is proportional to the square of the magnetization (M_s_^2^), which relates to the intrinsic superparamagnetism of the nanoplates and determines the strength of the local magnetic field inhomogeneities, as well as to the square of the effective radius of the magnetic core (R^2^), which determines the field perturbation areas for the outer-sphere protons. The rapid random flipping of the anisotropic nanoplates, when represented by an equivalent simulated sphere, generate a larger area of local field inhomogeneity compared with nanospheres under the same applied magnetic field. The balance of T_1_ and T_2_ contrasts was attained by controlling the structure and surface features of the nanoplates, including morphology (nanoplates vs. spheres in IOP-4.8@stPE) and surface coating (e.g., IOP-4.8 vs. IOP-4.8@SiO_2_ and IOP-4.8@stPE), with a large decrease in r_1_ due to blocking of the exposure of the facets causing the disappearance of the IS contribution and corresponding decrease in r_2_/r_1_, leading to change from DMCAs to T_2_ agents. The decrease in the nanoplate thickness decreased the r_2_/r_1_ value, as IOP-8.8 (r_2_/r_1_ ~ 8.18) is T_2_-dominated, while the IOP-4.8 (r_2_/r_1_ ~ 4.22) is a T_1_–T_2_ DMCA [89].

Table 4 shows that not all the systems described reach the low r_2_/r_1_ values recommended for efficient DMCAs [90,91].

3. Triggered aggregation change in ESIONs

The design of T_1_–T_2_ DMCAs can also be based on a different mechanism, based on the change in the aggregation state of extremely small-sized iron oxide nanoparticles (ESIONs) in the size range of 1.5–4 nm with relatively high r_1_ values, triggered by an external signal, such as pH change, a redox reaction or laser light. The aggregation of ESIONPs can generate T_2_ contrast effects due to the enhancement of magnetic field inhomogeneity and magnetic coupling between Fe centers. Some examples of such systems are summarized in Table 4. An example is based on pH-sensitive hydrazine functionalized ESIONPs forming assemblies (IONAs) cross-linked by small-molecular aldehyde derivative ligands. The dynamic formation and cleavage of hydrazone (–C=N–N–) (f) linkages in neutral and acidic environments, respectively, is the base for the reversible response of the nanoassemblies to pH variations. At pH 7.5, IONAs are stable and with high r_2_/r_1_ = 34.2, while at acidic pH 5.5, such as in the acidic tumor microenvironment (TME), the hydrazone bonds are cleaved and the IONAs are disassembled into hydrophilic dispersed ESIONs, with a r_2_/r_1_ = 4.1. The change in this relaxivity ration results from an increase in the number of second sphere water molecules (q_SS_) and a large decrease in the magnetization (M_s_) of the dispersed ESIONs relative to the IONAs, which affect their r_1_ and r_2_ values, respectively [92]. Another example is based on ESIONs linked with the targeting ligand folic acid (FA) binding arthritis-associated macrophage cells, and the light responsive diazirine (DA) through a PEG spacer (Fe_3_O_4_-PEG-(DA)-FA). These nanoparticles can form nanocomposites (NCs) upon laser irradiation to have tunable r_1_ and r_2_ values upon variation in the laser irradiation time. The change in the r_2_/r_1_ value from 2.36 in the NPs to 18.8 in the NCs led to the use of the designed Fe_3_O_4_-PEG-(DA)-FA NPs as T_1_ CAs in in vivo MRI of an arthritis mouse model without lasers and enhanced T_1_–T_2_ DMCAs in the arthritis inflamed region under laser irradiation due to the formation of NCs that accumulated within the arthritis region and their limited intravasation back to the blood circulation [93].

Again, Table 4 shows that not all the systems described reach the low r_2_/r_1_ values recommended for efficient DMCAs: while the first one does [92], the second one reaches quite high r_2_/r_1_ values [93]. Besides this, in some cases, T_1_/T_2_ triggering effects, such as pH decrease or reduction by GSH in the TME, led to final r_2_/r_1_ values below or above the range where T_1_–T_2_ DMCAs operate, forming instead T_1_/T_2_ switching MRI CAs [94,95,96,97,98,99,100,101]. This illustrates how the control of the switching mechanism at the molecular level is difficult.

**Table 4:** Summary of basic properties of the examples of NPs based on a typical *T*_2_ ((U)SPION)) contrast agent discussed in this section.

NPs Components	*r*_1_ (mM^−1^.s^−1^)	*r*_2_ (mM^−1^.s^−1^)	*r*_2_/*r*_1_	B_o_ (T)	d_H_ (nm); ζ (mV)	In Vitro/In Vivo Model for MRI	Ref.
USPION-PMAA-PTTM	8.3	35.1	4.2	4.7	3.3 (core); -*	In vitro phantoms /In vivo mice	[85]
USPION-PAA	8.20	16.67	2.03	7.0	1.7 (core): -*	In vivo Kunming mice	[86]
	6.15	28.62	4.65		4.6 (core); -*		
USPION-PAA	10.52	38.97	3.7	3.0	41.3; -14.7	In vivo rabbit	[87]
USPION-PEG	1.37	7.53	5.5	7.0	2.3 (core); d_H_ ≈12; -*	In vivo rat brain angiography (MRA)	[88]
Fe_3_O_4_ nanoplates:				0.5		In vitro phantoms	[89]
IOP-4.8	43.18	182.2	4.22		4.8 thickness		
IOP-4.8@SiO_2_	2.0	118.73	59.3		5.6 thickness		
IOP-4.8@stPE spheres	3.59	338.9	94.4		90; -*		
Fe_3_O_4_ nanocubes	5.23	89.68	17.1	3.0	27.8; -*	In vivo SD rats	[90]
ND-PEG-tNCIO	31.8	790.6	24.7		50; −61	In vitro phantoms	[91]
IONA → ESIONP dispersed pH 7.4 → 5.5.	3.2 → 108.0	5.1 → 22.3	34.2 → 4.1	3.0	80	In vitro A549 cells In vivo tumor mice	[92]
Fe_3_O_4_-PEG-(DA)-FA → NCs (Laser)	3.83 → 1.60	9.04 → 31.6	2.36 → 18.8	*	*	In vivo arthritis mouse model	[93]

* Not available.

#### 3.2.2. DMCAs including Both T_1_ and T_2_ Contrast Materials in the Same Nanoparticle

**Figure 4:**
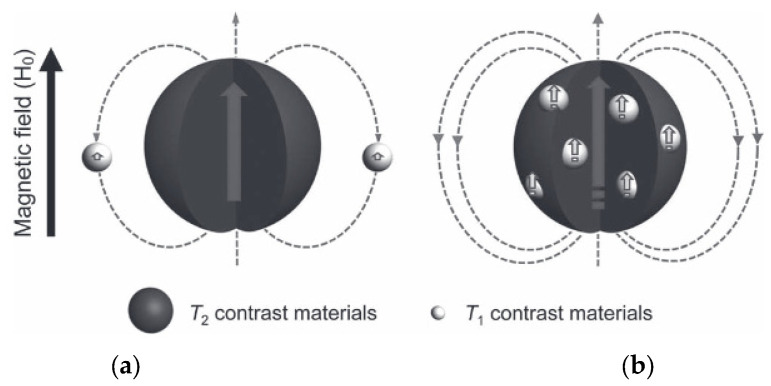
Two spin phenomena between T_2_ and T_1_ contrast materials with different locations. (**a**) Left—the local magnetic field intensity of T_1_ contrast materials is reduced when located outside of the T_2_ contrast material. (**b**) Right—The local magnetic field strengths of T_1_ and T_2_ contrast materials are enhanced simultaneously when T_1_ contrast materials are located inside the T_2_ contrast materials. Reproduced from [103].

The strategy of including both T_1_ and T_2_ contrast materials in the same nanoplatform has used several different designs to obtain hybrid nanostructures: (a) NPs integrating a superparamagnetic T_2_-contrasting material inside a paramagnetic T_1_ material (e.g., a Gd^3+^ complex); (b) doping a superparamagnetic NP with paramagnetic T_1_ contrast materials inside them; (c) T_1_ and T_2_ contrast materials connected side-by-side to form hybrid nano-oligomers (DB-HNT). In all these designs, with both contrast materials in the same nanoplatform, the magnetic fields generated by each CA disturb the relaxation process of the other. However, as typical paramagnetic T_1_ CAs have low r_1_ values compared to the r_2_ values of superparamagnetic T_2_ agents due to their relative magnetic moments, their effect on the T_1_ relaxation of the T_1_ contrast material is much larger than the opposite. The T_2_ agent generates a strong magnetic field induced by the external B_o_, which is dependent on 1/r^3^ (r = distance from the T_2_ agent), which affects the electronic spin relaxation time (T_1e_) of the paramagnetic T_1_ agent depending on their relative locations. The resulting effects of this magnetic coupling depends on the separation distance (d) between T_1_ and T_2_ agents [102] and also on their relative positions within the NP (Figure 4) [103].

1. NPs with a T_2_ material inside a paramagnetic T_1_ material

For systems consisting of a superparamagnetic core within a paramagnetic shell, the strong magnetic field from the core opposes the magnetic field created by the shell and reduces it (Figure 4, left), strongly quenching its r_1_ value. Their magnetic interaction is proportional to the inverse sixth power of their separation distance (d^−6^), as shown by a study where the distance-dependent magnetic resonance tuning (MRET) strategy for tuning the r_1_ value of a T_1_ agents was introduced [102]. A series of spherical NPs was designed, consisting of three components, where the paramagnetic enhancer (Gd-DOTA) was separated from the superparamagnetic quencher (a 12 nm Zn_0.4_Fe_2_._6_O_4_ NP) by controlling the thickness of a SiO_2_ separating layer decreasing from 18 to 2 nm (Zn_0.4_Fe_2_._6_O_4_@SiO_2_@ Gd-DOTA). A decrease in r_1_ was consistently observed as the separation distance between the T_1_ and T_2_ agents decreased. The T_1_ MRI signal was quenched when the d value between the enhancer and the quencher decreased and r_1_ decreased from 1.58 to 0.13 mM^−1^.s^−1^ (3 T), which was a consequence of the increase in the T_1e_ value of the T_1_ agent [102].

The strategy of including a layer of increasing thickness to increase the T_2_ core-T_1_ shell distance, and thus modulating their magnetic coupling, was pursued using not only inorganic porous materials like SiO_2_, but also micellar structures incorporating organic block copolymers and inorganic porous materials as possible frameworks [104]. The basic properties of several examples from the literature are summarized in Table 5. These involve the formation of spherical or cubic core–shell NPs integrating a superparamagnetic T_2_-contrasting core (e.g., Fe_3_O_4_, MnFe_2_O_4_) inside a paramagnetic T_1_ shell (e.g., Gd_2_O_3_, Gd_2_O(CO_3_)_2_, MnO) [105,106,107,108,109,110,111,112], or conjugating the superparamagnetic core with a paramagnetic Gd^3+^ or Mn^2+^ complex at its surface [113,114,115,116,117]. Generally, a sharp decrease in the magnetic coupling effect upon increasing the core–shell separation was verified experimentally by including a silica shell of increasing thickness between the two materials [105,106,107], or by increasing the distance between the core and the layer of pendant paramagnetic complexes through longer spacer groups [102,113]. Here, we will discuss only a few of the examples in more detail.

**Figure 5:**
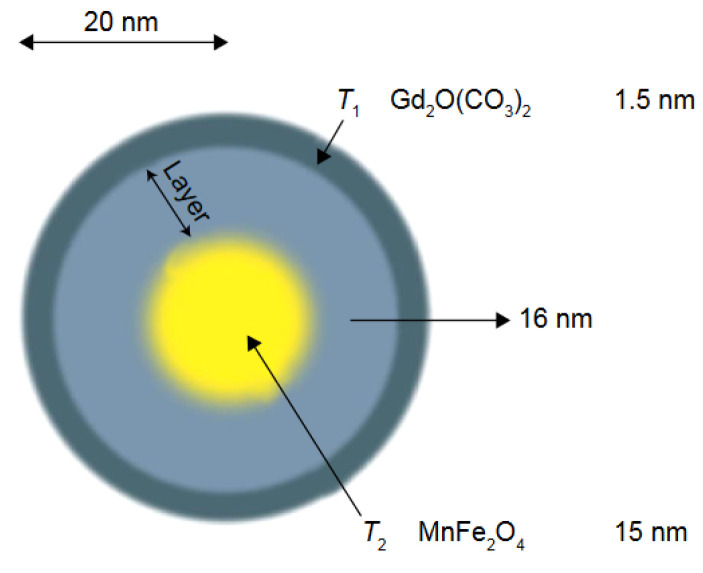
Schematic image of the core–shell-type dual-mode nanoparticle [MnFe_2_O_4_@SiO_2_@Gd_2_(CO_3_)_2_]. The T_1_ contrast material is positioned on the shell to have direct contact with the water for high T_1_ contrast effects, and the superparamagnetic contrast material is located at the core, inducing a long-range magnetic field for the relaxation of water. Reproduced from [31].

For instance, a core–shell-type T_1_–T_2_ DMCA agent has been described, where the T_1_ contrast material, a Gd_2_O(CO_3_)_2_ layer (1.5 nm thickness) was located on the shell in order to be in direct contact with water molecules, to obtain for high T_1_ contrast; the superparamagnetic T_2_ contrast material, MnFe_2_O_4_ (15 nm size) was located at the core, from where it could induce a long-range magnetic field for the relaxation of water molecules. The two materials were separated by a SiO_2_ layer of increasing (4, 8, 12, 16 and 20 nm) thickness (MnFe_2_O_4_@SiO_2_@Gd_2_O(CO_3_)_2_ NPs). As the SiO_2_ layer became thicker, the magnetic coupling decreased, the T_1_ quenching was reduced and r_1_ increased (from 2.0 to 32.5 mM^−1^.s^−1^) while the r_2_ decrease was weaker (332 to 213 mM^−1^.s^−1^). When the thickness of the SiO_2_ layer was equal of larger than 16 nm, the r_2_/r_1_ values decreased from 160 to 6.5, and the NPs became T_1_–T_2_ DMCAs, as both T_1_ and T_2_ effects became larger than the effects of the individual single-mode contrasts (Figure 5) [105].

**Figure 6:**
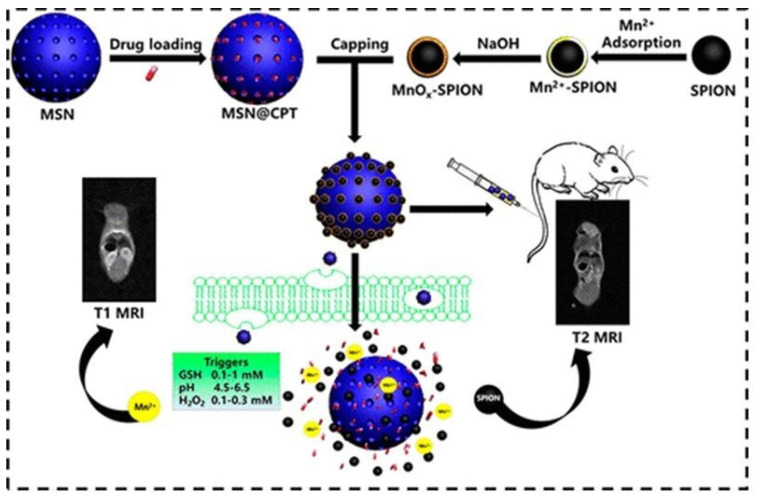
Schematic illustration for the synthesis of MnOx-SPION capped MSN and controlled drug release in response to the tumor microenvironment. In vivo T_2_ and T_1_ MRI images of a mouse upon injection of a MnOx-SPION@MSN solution through tail vein. Reproduced with permission from [111]. Copyright from the American Chemical Society.

A smart nanotheranostic system for early diagnosis and therapy of cancer was developed, consisting of camptothecin (CPT)-loaded mesoporous silica nanoparticles (MSN) capped with manganese oxide (MnO_x_)-coated SPIONs (MnO_x_-SPION@MSN@CPT NPs). The acid, oxidative stress and redox (GSH) response of MnO_x_ regulated the CPT drug release from the MSN channels, while the high magnetization of the surface SPIONs achieved high r_2_ values (102.2 mM^−1^.s^−1^). At the same time, degradation of the MnO_x_ shell caused release of Mn^2+^ in the TME, enhancing r_1_ (2 → 13.6 mM^−1^.s^−1^). The efficacy of this MRI responsive theranostic T_1_–T_2_ DMCA was confirmed in vitro on pancreatic cancer cells and in vivo on tumor-bearing mice (Figure 6) [111].

**Figure 7:**
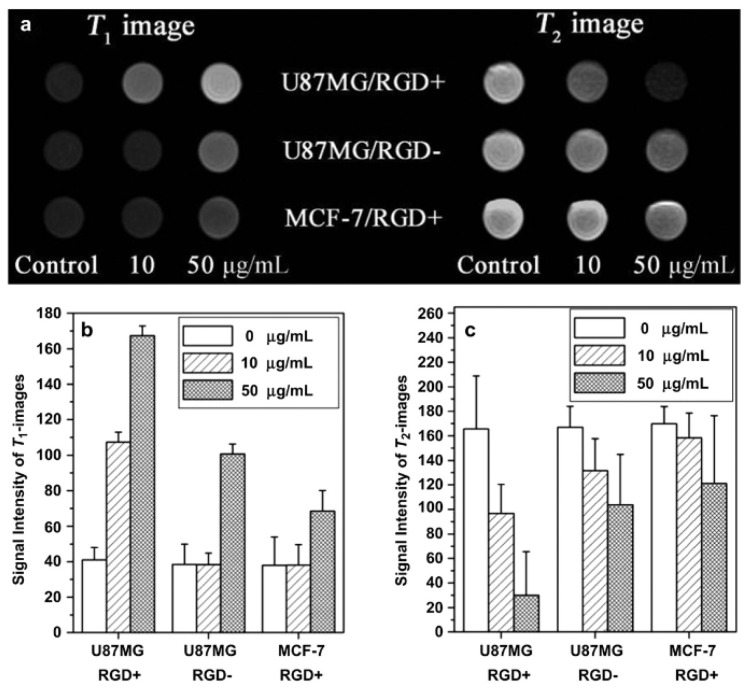
(**a**) T_1_- and T_2_-weighted MR images of Fe_3_O_4_@SiO_2_ (Gd-DTPA)-RGD NPs in U87MG and MCF-7 cells at different concentrations of NPs after incubation for 6 h on a 3T MRI system (RGD+: Fe_3_O_4_@SiO_2_ (Gd-DTPA)-RGD). RGD-: Fe_3_O_4_@SiO_2_(Gd-DTPA); (**b**) signal intensity analysis for T_1_-weighted MR images; (**c**) signal intensity analysis for T_2_-weighted MR images. Reproduced with permission from [117]. Copyright from Elsevier.

Some examples of systems conjugating the coating of SPIONs with a paramagnetic Gd^3+^ or Mn^2+^ complex will now be discussed briefly (Table 5) [113,114,115,116,117]. Up to now, the highest relaxivities for T_1_–T_2_ DMCAs are r_1_ = 31.6 mM^−1^.s^−1^and r_2_ = 836.7 mM^−1^.s^−1^ (at 1.47 T) which was obtained by Long et al. for Fe_3_O_4_@ALA-GdDOTA [113]. Superparamagnetic silica-coated iron oxide core–shell nanoparticles (Fe_3_O_4_@SiO_2_) conjugated at the surface to Gd-DTPA and the arginine-glycine-aspartic acid (RGD) peptide as a targeting ligand (Fe_3_O_4_@SiO_2_(Gd-DTPA)-RGD) NPs were synthetized. The NPs with a 21 nm diameter were water-dispersible, stable, and biocompatible, and with r_1_ = 4.2 mM^−1^.s^−1^ and r_2_ = 17.4 mM^−1^.s^−1^ (r_2_/r_1_ = 4.1) at the Gd/Fe molar ratio of 0.3:1. In vitro MRI experiments with U87MG and MCF- tumor cells over-expressing the high-affinity α_v_β_3_ integrin showed targeted T_1w_ positive and T_2w_ negative contrast when loaded with the NPs (Figure 7) as well as in in vivo U87MG tumor mice injected with the NPs, proving their potential as targeted T_1_–T_2_ DMCAs [117].

In other studies, core–shell NPs with different metal ions in the shell and/or in the core were studied (Table 5). Superparamagnetic SPION core−porous silica shell nanoparticles, containing the paramagnetic complexes ([Ln(btfa)_3_(H_2_O)_2_]) (btfa = 4,4,4-trifluoro-L-phenyl-1,3-butanedione, Ln = Gd/Eu) (γ-Fe_2_O_3_@SiO_2_/[Gd/Eu(btfa)_3_(H_2_O)_2_]) imbedded in the shell, with a 50 nm diameter and a 10 nm core, performed as a promising trimodal T_1_−T_2_ MRI and optical imaging contrast agent in Hela cells suspensions in vitro [118]. Also, colloidal suspensions of Fe/Fe_2_O_3_ NPs capable of providing both T_1w_ and T_2w_ MR images were synthesized [119]. Fe@Fe_3_O_4_-Gd(DOTA) NPs are strongly paramagnetic at room temperature with a M_s_ = 55 emu.g^−1^, and r_2_ and r_1_ values higher than those of Fe@Fe_3_O_4_ and Gd(DOTA) alone. These increased relaxivities suggest that the NPs are potential T_1_–T_2_ DMCAs for MRI [120]. Avocado-like Fe^3+^/Fe_2_O_3_ NPs were developed for working as T_1_–T_2_ DMCAs, based on a PDA-Fe^3+^-TA (tannic acid) coordination network (CNMN) embedded with DOX (Fe_2_O_3_@PDA-Fe^3+^-TA-CNMN/DOX). These NPs had suitable r_1_ and r_2_ values and the strong heat generated by Fe^3+^-TA allowed the CNMN to act as a photothermal agent and to effectively deliver the chemotherapeutic drug DOX to achieve chemo-photothermal combination therapy [121]. Finally, an iron core (with its subsequent oxidation giving a ferrite shell) with added Ni^2+^ ions to form a superparamagnetic nickel ferrite shell NP has been studied; with its surface treated with dopamine-PEG to make it dispersible, and it acts as a T_1_–T_2_ DMCA [122].

Table 5 shows that many of the systems described using this strategy have r_2_/r_1_ values above the values recommended for efficient DMCAs [107,109,110,113,115,118,120,121]. Besides this, the systems based on the release of toxic free Mn^2+^ in the TME, although interesting for animal studies, are not suited for clinical applications [110,111].

**Table 5:** Summary of basic properties of the examples of NPs with a T_2_ material inside a paramagnetic T_1_ material.

NPs Components	*r*_1_ (mM^−1^.s^−1^)	*r*_2_ (mM^−1^.s^−1^)	*r*_2_/*r*_1_	B_o_ (T)	d_H_ (nm); ζ (mV)	Therapeutic Modality	In Vitro/In Vivo Model for MRI	Ref.
MnFe_2_O_4_@SiO_2_@ Gd_2_O(CO_3_)_2_	2.0 → 32.5	332 → 213	166 → 6.5	4.7	26 → 58	-	In vitro phantoms	[105]
MnFe_2_O_4_@SiO_2_@ Gd_2_O(CO_3_)_2_	3.7 → 32.3	312 → 208	84.3 → 6.4	3.0	31 → 55	-	In vitro phantoms	[106]
Fe_3_O_4_@mSiO_2_/PDDA/BSA-Gd_2_O_3_	11.47	195.1	17.0	3.0	345.6; +26.9	-	In vitro 786-0 cells In vivo BALB/c mice	[107]
Fe_3_O_4_@Gd_2_O_3_ nanocubes	45.24	186.51	4.1	1.5	9.2; -*	-	In vitro phantoms In vivo SD rats (3 T)	[108]
Fe_3_O_4_@MnO-PEG	1.3	35.8	28	3.0	5.0; -*	-	In vivo BALB/c mice (7.0 T)	[109]
Fe_3_O_4_@Mn_3_O_4_ → Fe_3_O_4_ +Mn^2+^ (GSH)	2.4 → 16.1	92 → 258	38.4 → 16.1	1.5	22; -*	-	In vivo MKN-45 tumor-bearing mice	[110]
MnO_x_-SPION @MSN@CPT → SPION +Mn^2+^ (GSH, pH)	2 → 13.6	102.2 → -*	*	0.5	120; -*	ChT (CPT)	In vivo pancreatic-tumor- bearing mice	[111]
Fe_3_O_4_@ALA-GdDOTA (NP5) Fe_3_O_4_@ALA-Mn-DOTA (NP6)	31.6	836.7	26.4	1.47	6; *	-	In vitro phantoms	[113]
			
	14.2	324.5	22.8					
Fe_3_O_4_@DOPA-GdDTPA-PEG	11.17	30.32	2.7	3.0	73.8; −5.5	-	In vivo BALB/c nude mice.	[114]
Fe3O4/CuInS_2_@SiO2- (GdDTPA)-RGD	1.56	23.22	14.9	3.0	45; +8.16	-	In vivo BSPC-3 pancreatic tumor mice	[115]
MnFe_2_O_4_@Gd: FA-DTPA-PEG-DIB-	20.59	68.48	3.32	0.55	18; *	-	In vitro Hela and 3T3 cells	[116]
Fe_3_O_4_@SiO_2_-GdDTPA-RGD	4.2	17.4	4.1	3.0	27; +7.25	-	In vitro U87MG cells In vivo U87MG tumor mice	[117]
γ-Fe_2_O_3_@SiO_2_/[Gd/Eu (btfa)_3_(H_2_O)_2_]	1.0	75.9	78	9.4	50; −40	-	In vitro Hela cells	[118]
Fe@Fe_3_O_4_-GdDOTA	7.2	109.4	15.2	0.5	358; +24.6	-	In vitro 4T1 cells In vivo 4T1 tumor mice	[120]
Fe_2_O_3_@PDA-Fe^3+^-TA-CNMN/DOX	5.01	125.45	25.0	7.0	95.6; −30.3	ChT(DOX)/ PTT	In vivo tumor mice	[121]
Fe@NiFe_2_O_4_-PEG/dopamine	7.19	9.96	1.4	2.4	10–15; *	-	In vitro phantoms	[122]

* Not available.

2. Doping superparamagnetic T_2_ contrast NPs with paramagnetic T_1_ contrast materials inside

**Figure 8:**
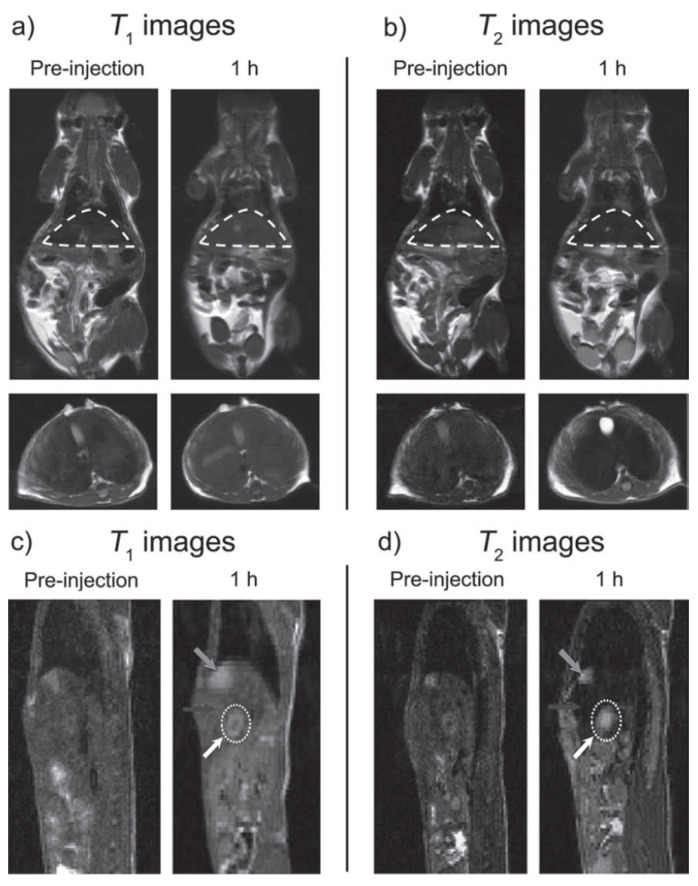
Simultaneous T_1_ and T_2_ MRI imaging of liver and hepatic tumor (7 T), respectively. (**a**) T_1w_ and (**b**) T_2w_ in vivo MR images of BALB/c mice (**top**: coronal plane, **bottom**: transverse plane) before and after iv. injection of GdIO nanoparticles with a dose of 2.0 mg kg^−1^. The regions of liver in the coronal planes were circled by dash lines. (**c**) T_1w_ and (**d**) T_2w_ in vivo MR images of nude mice orthotopically inoculated with HepG2 liver cancer cells (sagittal plane) before and after iv. injection of GdIO nanoparticles with a dose of 2.0 mg Fe kg^−1^. Grey arrows: gallbladder, black arrows: liver, white dotted circles and white arrows: liver tumor. Reproduced from [103].

For systems where the paramagnetic material resides inside the superparamagnetic iron oxide, the magnetic fields of both materials reinforce each other simultaneously, strongly enhancing the r_1_ value and causing a synergistic T_1_–T_2_ enhancement effect (Figure 4, right) [103]. The basic properties of systems using this strategy are shown in Table 6, some of which are discussed in some detail. The theory of synergistic T_1_–T_2_ enhancement effect discussed above was confirmed by Gao et al. using Gd_2_O_3_-embedded Fe_3_O_4_-HDA-G_2_ NPs (GdIO-HDA-G_2_), which showed a synergistic enhancement of r_1_ and r_2_. The GdIO had higher r_2_ (146.5 mM^−1^.s^−1^) than Fe_3_O_4_ (125.4 mM^−1^.s^−1^) of similar size and also higher r_1_ (69.5 mM^−1^.s^−1^) than Gd_2_O_3_ (12.1 mM^−1^.s^−1^) of similar size. Furthermore, the Gd_2_O_3_ NPs showed no enhanced T_2_ contrast, while Fe_3_O_4_ nanoparticles showed limited enhanced T_1_ contrast. Simultaneous in vivo T_1w_ and T_2w_ MRI of BALB/c mice upon i.v. injection of GdIO showed simultaneous strong MRI contrast enhancement of liver in both types of images due to the high accumulation of NPs in the hepatic Kupffer cells of the liver mononuclear phagocyte system (MPS). The same MRI experiment in HepG2 tumor mice detected the liver lesions through pseudo-negative and pseudo-positive contrast effects because the contrast between lesions and surrounding normal liver tissue increased due to the very low uptake by hepatic tumors, which contain few active Kupffer cells and macrophages (Figure 8). This work validated the new strategy for the design of new T_1_–T_2_ DMCAs [103].

Another example of the use of the same design strategy consisted of water-dispersible GdIO NPs stearic acid modified low molecular weight polyethyleneimine (stPEI) (GdIO–stPEI). This nanoplatform was capable of binding and delivering siRNA for gene knockdown and work as T_1_–T_2_ DMCA with HCT-116 cells in vitro [123]. Zwitterion dopamine sulfonate-coated superparamagnetic GdIO NPs (GdIO-ZDS) with small core size (2.8–4.8 nm) showed partial paramagnetism at room temperature, with a decreased M_S_ value relative to IOs of the same core size. This resulted from the combination of surface canting with the effect of the embedded Gd_2_O_3_ nanoclusters which disturbs the long-range order of magnetic spins in the small GdIO NPs. It also led to a significantly increased r_1_ value and decreased r_2_ value relative to IO-ZDS of the same core size. For example, GdIO NPs with a 4.8 nm diameter, had a high r_1_ = 7.85 mM^−1^.s^−1^ and a low r_2_/r_1_ = 5.24 relative to IO (r_2_/r_1_ = 9.59). These NPs caused a strong positive tumor contrast effect in T_1w_ MRI images of SKOV3 human ovarian tumor mice through an enhanced permeation and retention (EPR) effect [124].

Superparamagnetic Fe_3_O_4_ NPs have also been doped with other paramagnetic ions, such as Mn^2+^ (MnIO) and Eu^3+^ (EuIO) [125,126]. The MRI contrast abilities of uniform Mn^2+^-doped iron oxide (MnIO) NPs nanoparticles with 5, 7, 9 and 12 nm size were studied. The NPs were superparamagnetic at 300 K, with M_s_ values which decreased with the decrease in the MnIO NP sizes, from 71.0 emu g^−1^ for the 12 nm NPs to 39.7 emu g^−1^ for the 5 nm NPs due to the spin canting effect at their surface. Their r_1_ and r_2_ values were highly size-dependent, with r_2_/r_1_ values decreasing from 7.4 for the 12 nm NPs to 2.6 for the 5 nm NPs. Thus, by controlling the size of the MnIO NPs, T_1_-dominated, T_2_-dominated, and T_1_–T_2_ DMCAs could be obtained with much higher contrast enhancement than the corresponding conventional iron oxide nanoparticles, as verified by in vivo MRI of BALB/c mice [125]. Finally, Eu^3+^-dopped iron oxide nanocubes (EuIO) were developed as T_1_–T_2_ DMCAs for in vivo MRI. The EuIO nanocubes were composed of mixed Fe_3_O_4_ (magnetite) and Eu_2_O_3_ nanoclusters of 10.0, 14.0 and 20.1 nm size, due to the large ionic radius of Eu^3+^ ions (94.7 pm) which prevented them from occupying either the tetrahedral or the octahedral interstitial sites in the spinel structure. The EuIO nanocubes are partially paramagnetic at 300 K, which is different from the superparamagnetism of magnetite NPs, due to increased spin canting on their surface layer after Eu_2_O_3_ embedding. The Eu_2_O_3_ clusters located inside the iron oxide NPs nanoparticles disturbed the local magnetic field intensity of the whole NPS and reduced their M_s_ values (~39.6 emu g^−1^) relative to magnetite NPs with a similar size (~53.4 emu g^−1^) at 300 K. The larger EuIO NPs had higher M_s_ values due to the loss of the spin canting effect on the particle surface. As a result, both r_1_ and r_2_ values of EuIO nanocubes could be tuned by varying their sizes and Eu doping ratios. Larger EuIO nanocubes had higher r_1_ and r_2_ values. The Eu/Fe molar ratio also had an important role in the r_1_ and r_2_ values of EuIO nanocubes: raising the Eu molar ratio increased r_1_ due to the spin order of Eu^3+^ ions which had the same orientation as the local magnetic field, while it decreased r_2_ values due to the reduction in the M_s_ values after Eu embedding. For instance, EuIO nanocubes of 14 nm diameter showed a high r_1_ = 36.8 mM^−1^.s^−1^, which is approximately 3 times higher than that of Fe_3_O_4_ NPs (12.47 mM^−1^.s^−1^) of similar size. After citrate coating, EuIO nanocubes produced enhanced T_1_ and T_2_ MRI contrast effects in Sprague Dawley rats as models for in vivo MRI studies, in particular in the cardiac and liver regions [126].

In summary, Table 6 shows that the r_2_/r_1_ values of the composite NPs (in bold) are more suitable as DMCAs when compared with those of the NPs made of their components.

3. T_1_ and T_2_ contrast materials connected side-by-side forming hybrid oligomers of different shapes

**Figure 9:**
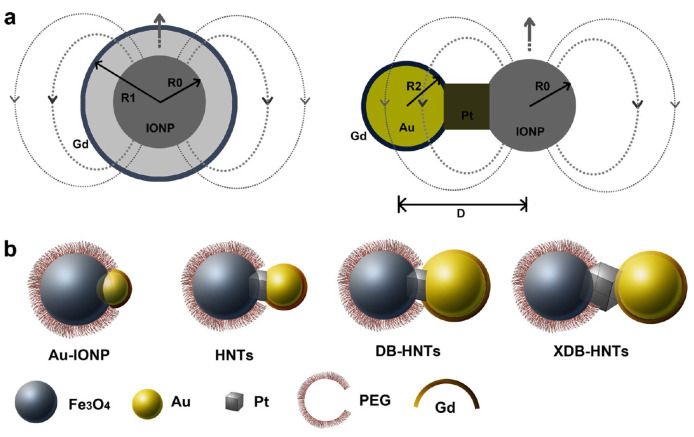
(**a**) Engineering the heterogeneous nanostructures for magnetic coupling of T_1_ and T_2_ contrast agents with (**left**) core/shell structures or (**right**) dumbbell structures. R_0_ is the radius of T_2_ contrast agent (iron oxide nanoparticles), R_1_ is the radius with the Gd shell, R_2_ is the radius of gold nanoparticles, and D represents the center-to-center distance between iron oxide and gold nanoparticles. (**b**) Design and characterization of dumbbell heterostructures for dual T_1_ and T_2_-weighted MRI. Illustration of constructions of four different types of dumbbell-like or dumbbell heterostructures (Au_IONPs, HNTs, DB-HNTs, and extra-large DB-HNTs (XDB-HNTs)). Adapted from [127].

A third strategy to control the interference by magnetic coupling between T_1_ and T_2_ materials present in a single-composite nanostructure is to engineer the architecture of heterogeneous NPs forming hybrid trimers and oligomers of different geometries, like dumbbell-shaped NPs [109,127] or nanoflowers [109] (Table 6). Dumbbell-shaped NPs, or so-called ‘Janus’ NPs, were synthesized, with two different components within one single structure, as solid-state analogues of bifunctional organic molecules to construct hybrid nanotrimers (HNTs) (Figure 9a, right panel), in which iron oxide and Au nanocrystals were connected by a platinum nanocube (Au-IONP). The surface of its Au component was covalently immobilized with Gd- cystamine-DOTA_2_ (dithiol derivative of DOTA) as a T_1_ material (Gd(DOTA)-HNTs). To reduce its magnetic coupling with the IONP (T_2_ material), the size of the Au nanocrystals was increased by controlling the seed-mediated growth processes during the synthesis and the size of the Pt cubes was also increased to increase the distance (D, Figure 9a, right panel) between the IONP and the Au nanocrystals. The resulting heterotrimers with large Au crystals had a dumbbell structure, (Gd(DOTA)-DB-HNTs and Gd(DOTA)-XDB-HNTs, the latter with a larger Au component and thus a larger number of Gd per single NP). The IO component was covered with PEG chains to make the whole NPs water-soluble and biocompatible (Figure 9b). The Gd(DOTA)-DB-HNTs and Gd(DOTA)-XDB-HNTs had increased r_1_ values due to the reduced magnetic coupling between the T_1_ and T_2_ components as their D values increased. Their r_2_ values were similar due to the similar sizes and shapes of the iron oxide components. The calculation of the r_1_ relaxivity of each particle was based on different concentrations of Gd: r_1_ (mM [Fe + Gd]^−1^.s^−1^) or r_1_′ (mM [Gd]^−1^.s^−1^). Even though the r_2_/r_1_ ratio of Gd(DOTA)-DB-HNTs was 33, their r_2_/r_1_´was only 4.2, indicating that they could be T_1_–T_2_ DMCAs. Although Gd(DOTA)-XDB-HNTs have slightly higher r_1_′ (32.1 mM [Gd]^−1^.s^−1^), they did not have a better r_2_/r_1_´ ratio (4.2) than Gd(DOTA)-DB-HNTs because of their larger size of the IO component, which increased r_2_. The hybrid heterotrimers were highly stable in physiological conditions and induced simultaneous positive and negative contrast enhancements in in vivo MRI images of HT-29 tumor-bearing mice upon i.v. injection of Gd(DOTA)-DB-HNTs, showing their potential as T_1_–T_2_ DMCAs [127].

**Figure 10:**
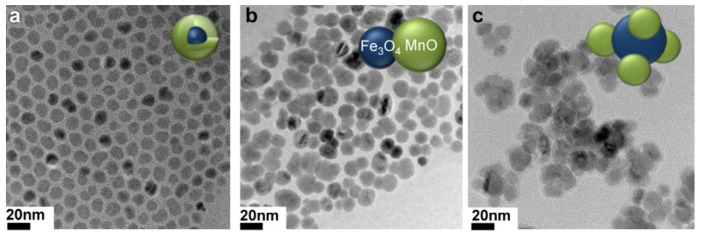
TEM images of (**a**) Fe_3_O_4_ (5 nm)@MnO, (**b**) Fe_3_O_4_ (11 nm)/MnO dumbbell-like, and (**c**) Fe_3_O_4_ (21 nm)/MnO flower. Reproduced with permission from [109]. Copyright from Elsevier.

**Figure 11:**
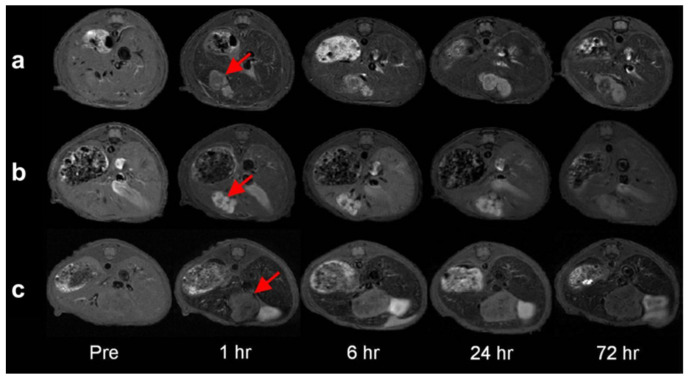
Serial T_2_-weighted MR images of the orthotopic xenograft model of a nude mouse liver for human HCC (arrows) before as well as 1 h, 6 h, 24 h and 72 h after the contrast injection of (**a**) Fe_3_O_4_/MnO dumbbell nanocrystals, (**b**) MnO nanocrystals, and (**c**) and Fe_3_O_4_ nanocrystals. Reproduced with permission from [109]. Copyright from Elsevier.

Fe_3_O_4_/MnO hybrid nanocrystals were prepared based on seed-mediated growth of MnO on the surface of a Fe_3_O_4_, where the resulting structures depended on the size of the seed NP, producing core/shell spherical Fe_3_O_4_ (5 nm)@MnO, dumbbell shaped Fe_3_O_4_ (11 nm)/MnO, and flower-shaped Fe_3_O_4_ (21 nm)/MnO hybrid NPs from 5 nm, 11 nm, and 21 nm Fe_3_O_4_ NPs, respectively (Figure 10). All the NPs had a high r_2_/r_1_ ratio and the dumbbell shaped Fe_3_O_4_/MnO NPs produced a negative contrast effect in T_2_^*^_w_ MRI images and a positive contrast effect in T_1w_ MRI images upon releasing Mn^2+^ ions in a low pH environment, in vitro (aqueous phantoms) and in vivo (normal brain of a nude mouse) obtained after i.v. injection. The same study using an orthotopic xenograft model of human hepatocellular carcinoma (HCC) showed high contrast between relatively hyperintense HCC and hypointense background liver parenchyma in T_2w_ MRI HCC and hypointense background liver parenchyma due to the presence of Kupffer cells in the later, while in T_1w_ MRI images a bright signal of the HCC tumor was observed after injection, due to HCC cell uptake of the Mn^2+^ ions liberated from the NPs in the low pH environment of the TME (Figure 11). This induced organ-specific contrast enhancement showed the potential of the hybrid NPs and T_1_–T_2_ DMCAs [109].

In summary, the synthetically challenging strategy of designing DMCAs based on forming hybrid oligomers of different shapes using T_1_ and T_2_ contrast materials, although scientifically original, has so far produced nanosystems with too high r_2_/r_1_ values [109,127].

## 4. General Issues of In Vivo Use of NPs as MRI Contrast Agents

Besides the problems of design and contrast efficacy described in previous sections, the use of MNPs as MRI CAs in vivo involves many other issues, including their colloidal stability, biocompatibility, absorption, distribution, metabolism, and excretion (ADME) [128], toxicity, targeting and potential theranostic use.

Several intrinsic properties of MNPs, such as size, shape, coating, charge and presence of surface ligands, influence their biodistribution, elimination and target site accumulation. MNPs should be colloidally stable in aqueous media and in body fluids, as in vivo agglomeration and precipitation causes unacceptable safety issues and limits their high-performance MRI function. The colloidal stability is ensured by high zeta potentials and coating with hydrophilic and biocompatible ligands, such as PEG, which also maintains their non-toxicity. The biocompatibility of the materials used in DMCAs is also quite variable. Fe^3+^-based NPs are more biocompatible than those containing Gd^3+^, Dy^3+^, Ho^3+^, Tb^3+^ and Mn^2+^, because iron is an essential element in the human body, participating in a wide variety of metabolic processes, including oxygen transport, DNA synthesis, and electron transport. However, its concentration in body tissues must be tightly regulated because in excessive amounts, Fe^2+^ is toxic through the generation of reactive oxygen species (ROS) via the so-called Fenton reaction, which eventually results in cell damage, cell death, and organ failure, primarily affecting the liver, heart, pancreas, thyroid, and central nervous system [129]. Although Mn is also an essential trace element, excessive doses of Mn^2+^ can be neurotoxic, and its build-up in the brain may lead to manganism, a neurological disorder similar to Parkinsonism [130]. Also, free Gd^3+^ ions liberated from Gd chelates or NPs into the body could cause nephrogenic systemic fibrosis (NSF) [14,15]. Non-toxicity of metal-based NPs is critical for their in vivo use as MRI CAs [131].

Because MRI CAs are usually intravenously injected, the preferable excretion of MNPs is through the renal system rather than the hepatobiliary pathway because the later one is relatively slow and the MNPs could be taken up by the liver reticuloendothelial system (RES) and be partially decomposed during the excretion process, which would be toxic to the body. This metabolization process of small (<10 nm) NPs can occur through direct penetration of the cell membrane (phagocytosis) and delivery to the cytoplasm and nucleus, while for larger NPs (10–200 nm) the uptake is mediated by clathrin-dependent endocytosis- Both mechanisms facilitate the cell lysosomal internalization and degradation of the MNPs [128].

For renal excretion, MNPs should be ultrasmall with hydrodynamic diameters less than 5 nm because the glomerular filtration diameter in the kidneys is 4.5–5 nm [132]. The kinetic stability of MNPs should also be high to avoid their decomposition until they are excreted as urine through the renal system.

In summary, MNPs as MRI CAs for safe in vivo applications should be kinetically stable (i.e., no decomposition), coated with hydrophilic and biocompatible polymers for non-toxicity and colloidal stability, and ultrasmall with hydrodynamic diameters less than 5 nm for renal excretion. This excretion route, avoiding as far as possible their liver retention in the hepatobiliary pathway, is also very important for targeting and theranostic drug delivery by MNPs [133,134]. In this review, several examples of in vivo use of theranostic DMCAs were presented, e.g., using ferroptosis inhibition [78], immunotherapy [79] and photodynamic therapy (PTT) associated with chemotherapy (ChT) [82] and chemodynamic therapy (CDT) [84]. As far as nanocarriers for drug delivery and theranostics are concerned, the inappropriate release, internal instability and tissue non-targeting effects, sometimes observed due to biological barriers, are the main restrictions for their in vivo application [128,135].

## 5. Conclusions

This review describes recent developments and optimization of MNPs containing Gd, Mn, Fe and other lanthanide ions as potential dual-mode T_1_–T_2_ MRI contrast agents (DMCAs). Their high performance was highlighted by describing selected in vivo MRI studies. However, the development stage of most of the reported MNP-based MRI CAs is still quite limited, both in vitro and in preclinical in vivo small animal studies. To improve their chances to reach the clinical trials stage, several key issues must be solved. These include long-term colloidal stability in aqueous media, toxicity and pharmacokinetics, which depend on their coating with hydrophilic and biocompatible ligands. In comparison with MNPs containing Mn, or lanthanides (such as Gd, Dy, Ho, and Tb), Fe-based NPs are less toxic because iron is an essential element, and several IONPs have been approved and commercialized as MRI CAs, such as Feridex^®^, Sinerem^®^, and Resovist^®^, before being discontinued due to lack of commercial interest. The understanding of the correlation between the physicochemical properties of MNPs and their biological behavior in vivo must also be improved using appropriate preclinical small animal studies.

Another major problem faced by the clinical introduction of new MRI CAs in general, and MNP-based MRI CAs in particular, due to their molecular complexity and the consequent expensive synthesis, is to solve the main regulatory challenges of controlled NPs synthesis, uniformity, batch-to-batch reproducibility and upscaling of production. This leads to large development costs, especially for phase III studies, compared with the post-approval revenues [136,137]. However, the large amount of information obtainable with molecular imaging using MNP-based MRI CAs, such as those described in this review, may contribute to early diagnosis and personalized therapies, which ultimately will reduce health care costs.

## Figures and Tables

**Table 2:** Main chemical and physical characteristics of paramagnetic ions used in MRI CAs.

Metal Ion	Free Ion Ground State/Configuration	Spin-Only Magnetic Moment (μB)	Ionic Radius (Å)	Coordination Number	Electronic Relaxation Times (s)	M-H_w_ Distance (Å)
Mn^2+^	^6^S_5/2_ (3d^5^)	5.9	0.83/0.90	6/7 (high spin)	10^−10^–10^−8^	~2.8
Fe^3+^	^6^S_5/2_ (3d^5^)	5.9	0.64	6–7	10^−11^–10^−9^	2.6–2.7
Eu^3+^	^7^F_0_ (4f^6^) (^5^D_0_, ^5^D_1_)+	3.4 *	1.06/1.12	8/9	10^−11^–10^−9^	~3.0
Gd^3+^	^8^S_7/2_ (4f^7^)	7.9	1.05/1.11	8/9	10^−10^–10^−8^	~3.0
Tb^3+^	^7^F_6_ (4f^8^)	9.7	1.04/1.10	8/9	10^−13^–10^−12^	~3.0
Dy^3+^	^6^H_15/2_ (4f^9^)	10.6	1.03/1.08	8/9	10^−13^–10^−12^	~3.0
Ho^3+^	^5^I_8_ (4f^10^)	10.4	1.02/1.07	8/9	10^−13^–10^−12^	~3.0

* The magnetic moment of Eu^3+^ is not zero due to the contribution of the excited states ^5^D_0_ and ^5^D_1_.

**Table 3:** Summary of basic properties of the examples of DMCA NPs based on a typical T_1_ contrast agent discussed in this section. Some of the systems in this Table have been used as theranostic agents. * Not available.

NPs Components	*r*_1_ (mM^−1^.s^−1^)	*r*_2_ (mM^−1^.s^−1^)	*r*_2_/*r*_1_	B_o_ (T)	d_H_ (nm); ζ (mV)	Therapeutic Modality	In Vitro/In Vivo Model for MRI	Ref.
Gd-DOTA@CH/HA hydrogel	72.3	177.5	2.5	1.5	260; -*	-	phantoms	[73]
Gd_2_O_3_@PASA	19.1	53.7	2.8	3.0	12.7; −28.0	-	in vivo mice	[74]
Gd_2_O_3_@G4.5 PAMAM-PEG	53.9	182.8	3.4	7.0	50.4; −0.2	-	in vitro RAW264.7 macrophages in vivo BALB/c female mice	[75]
Gd-DOTA-D-BSA	7.7	44.1	5.7	7.0	45; -*	-	ICR mice	[76]
MnO@DMMS	10.1	169.7	16.8	3.0	199; -		SD rats	[77]
PFOB@MnO_x_	0.41 → 6.13	5.39 → 43.75	15.0 → 8.1	*	170; -*	ferroptosis inhibition	tumor-bearing mice	[78]
R848@BNN@Mn-MPDA	10.2	129.3	12.7	7.0	201.4; -*	NO gas and immunotherapy	4T1 tumor-bearing mice	[79]
(Gd/Dy)_2_O_3_ @ D-glucuronic acid	*	*	6.6	*	1.0; -*	-	*	[80]
NaGdF4:Dy@DSPE-PEG_2000_	5.17	12.26	2.4	9.4	*	-	in vivo mice	[81]
Fe-PDA@CP3-DOX	7.52	45.92	6.1	1.5	144; -*	PTT/ChT	in vivo nude mice	[82]
Fe−NCP-BSA	5.3	10.9	2.1	7.0	97; −31.2	-	GL261 tumor-bearing mice	[83]
Fe-BPNS	0.84 → 6.19	20.5 → 154.8	24.4 → 25.0	3.0	208; −9.86	PTT/CDT	4T1 tumor-bearing mice	[84]

**Table 6:** Summary of basic properties of NPs made of a paramagnetic T_1_ material inside a superparamagnetic T_2_ material or forming hybrid oligomers of different shapes (in bold). Their properties are compared with those of the NPs made of their components.

NPs Components	*r*_1_ (mM^−1^.s^−1^)	*r*_2_ (mM^−1^.s^−1^)	*r*_2_/*r*_1_	B_o_ (T)	d_H_ (nm); ζ (mV)	In Vitro/In Vivo Model for MRI	Ref.
**Gd_2_O_3_@ Fe_3_O_4_-HDA-G_2_ (GdIO)**	69.5	146.5	2.1	0.5	14; -*	In vitro HeLa, HepG2 cells In vivo Balb/c/HepG2 tumor mice (7 T)	[103]
Fe_3_O_4_	*	125.4	*		14; -*		
Gd_2_O_3_	12.1	*	*		2; -*		
**GdIO–stPEI**	61.67	181.49	2.94	0.5	153.2; -15	In vitro HCT-116 cells	[123]
**GdIO–ZDS**	7.85	41.14	5.24	7.0	4.8 (core) 6.50; -*	In vivo SKOV3 tumor mice	[124]
**GdIO–ZDS**	4.63	34.38	7.43		3.5 (core) 5.61; -*		
**GdIO–ZDS**	3.05	26.45	8.56		2.8 (core) 4.18; -*		
IO@ZDS	6.14	58.94	9.59		4.9 (core) 7.13; -*		
**MnIO-tartarate**	18.0	45.9	2.6	0.5	5 (core) 10.49; -*	In vivo BALB/c mice (7 T)	[125]
	27.2	146.5	5.4		7 (core) 13.21; -*		
	32.1	205.5	6.4		9 (core) 16.79; -*		
	38.2	280.8	7.4		12 (core) 22.28; -*		
**EuIO-citrate**	36.79	97.52	2.65	0.5	14.0; -*	In vivo SD rats (3.0 T)	[126]
Fe_3_O_4_	12.47	116.78	9.36		14.0; -*		
Eu_3_O_4_	0.03	5.44	181		14.0; -*		
**GdDOTA-Au-IONPs**	1.65	123	74.5	7.0	19.5; −13.4	In vitro phantoms In vivo HT-29 tumor mice	[127]
**GdDOTA-HNTs**	1.74	131	75.2		21.2; −15.1		
**GdDOTA-DB-HNTs**	3.88	128	33.0		24.6; −16.0		
**GdDOTA-XDB-HNTs**	4.12	125	30.3		26.8; −16.4		
**Fe_3_O_4_@MnO-PEG sphere**	1.3	35.8	28	3.0	5; -	In vitro phantoms In vivo nude mice (brain) In vivo HCC nude mouse (liver) (7 T)	[109]
**Fe_3_O_4_/MnO-PEG dumbbell**	1.4	78.9	56		11; -*		
**Fe_3_O_4_/MnO-PEG flower**	0.6	141	235		21; -*		
Fe_3_O_4_-PEG sphere	0.8	152	190		5; -*		
Fe_3_O_4_-PEG sphere	1.1	162	147		11; -*		
Fe_3_O_4-PEG_ sphere	1.1	252	229		21; -*		

* Not available.

## References

[1] Reimer P., Parizel P.M., Meaney J.F.M., Stichnoth F.A. (2010). Clinical MR Imaging, a Practical Approach.

[2] Weissleder R., Mahmood U. (2001). Molecular imaging. Radiology.

[3] Caravan P., Ellison J.J., McMurry T.J., Lauffer R.B. (1999). Gadolinium (III) Chelates as MRI Contrast Agents:  Structure, Dynamics, and Applications. Chem. Rev..

[4] Merbach A.E., Helm L., Tóth É. (2013). The Chemistry of Contrast Agents in Medical Magnetic Resonance Imaging.

[5] Wahsner J., Gale E.M., Rodríguez-Rodríguez A., Caravan P. (2019). Chemistry of MRI Contrast Agents: Current Challenges and New Frontiers. Chem. Rev..

[6] Aime S., Botta M., Terreno E. (2005). Gd(III)-Based Contrast Agents for MRI. Adv. Inorg. Chem..

[7] Geraldes C.F.G.C., Laurent S. (2009). Classification and basic properties of contrast agents for magnetic resonance imaging. Contrast Media Mol. Imaging.

[8] Weissleder R., Pittet M.J. (2008). Imaging in the era of molecular oncology. Nature.

[9] Amoroso A.J., Pope S.J.A. (2015). Using lanthanide ions in molecular bioimaging. Chem. Soc. Rev..

[10] Villaraza A.J., Bumb A., Brechbiel M.W. (2010). Macromolecules, Dendrimers, and Nanomaterials in Magnetic Resonance Imaging: The Interplay between Size, Function, and Pharmacokinetics. Chem. Rev..

[11] Laurent S., Forge D., Port M., Roch A., Robic C., Vander Elst L., Muller R.N. (2008). Magnetic Iron Oxide Nanoparticles: Synthesis, Stabilization, Vectorization, Physicochemical Characterizations, and Biological Applications. Chem. Rev..

[12] Lee N., Hyeon T. (2012). Designed synthesis of uniformly sized iron oxide nanoparticles for efficient magnetic resonance imaging contrast agents. Chem. Soc. Rev..

[13] Lee N., Yoo D., Ling D., Cho M.H., Hyeon T., Cheon J. (2015). Iron Oxide Based Nanoparticles for Multimodal Imaging and Magnetoresponsive Therapy. Chem. Rev..

[14] Sherry A.D., Caravan P., Lenkinski R.E. (2009). Primer on gadolinium chemistry. J. Magn. Reson. Imaging.

[15] Thomsen H.S., Morcos S.K., Almén T., Bellin M.-F., Bertolotto M., Bongartz G., Clement O., Leander P., Heinz-Peer G., Reimer P. (2013). Nephrogenic systemic fibrosis and gadolinium-based contrast media: Updated ESUR Contrast Medium Safety Committee guidelines. Eur. Radiol..

[16] Thomsen H.S. (2009). Nephrogenic Systemic Fibrosis: History and Epidemiology. Radiol. Clin. N. Am..

[17] Berstein E.J., Schmidt-Lauber C., Kay J. (2012). Nephrogenic systemic fibrosis: A systemic fibrosing disease resulting from gadolinium exposure. Best Pract. Res. Clin. Rheumatol..

[18] Gianolio E., Bardini P., Arena F., Stefania R., Di Gregorio E., Iani R., Aime S. (2017). Gadolinium Retention in the Rat Brain: Assessment of the Amounts of Insoluble Gadolinium-containing Species and Intact Gadolinium Complexes after Repeated Administration of Gadolinium-based Contrast Agents. Radiology.

[19] Khairinisa M.A., Ariyani W., Tsushima Y., Koibuchi N. (2021). Effects of gadolinium deposits in the cerebellum: Reviewing the literature from in vitro laboratory studies to in vivo human investigations. Int. J. Environ. Res. Public Health.

[20] Wang Y.X.J., Idée J.-M. (2017). A comprehensive literature update of clinical researches of superparamagnetic resonance iron oxide nanoparticles for magnetic resonance imaging. Quant. Imaging Med. Surg..

[21] Doane T.L., Burda C. (2012). The unique role of nanoparticles in nanomedicine: Imaging, drug delivery and therapy. Chem. Soc. Rev..

[22] Helm L. (2006). Relaxivity in paramagnetic systems: Theory and mechanisms. Prog. Nucl. Magn. Reson. Spectrosc..

[23] Norek M., Peters J.A. (2011). MRI contrast agents based on dysprosium or holmium. Prog. Nucl. Magn. Reason. Spectrosc..

[24] Bertini I., Luchinat C., Parigi G., Ravera E. (2017). NMR of Paramagnetic Molecules.

[25] Gueron M. (1975). Nuclear relaxation in macromolecules by paramagnetic ions: A novel mechanism. J. Magn. Reson..

[26] Vega A.J., Fiat D. (1976). Nuclear relaxation processes of paramagnetic complexes-The slow-motion case. Mol. Phys..

[27] Roch A., Muller R.N., Gillis P. (1999). Theory of proton relaxation induced by superparamagnetic particles. J. Chem. Phys..

[28] Vuong Q.L., Berret J.-F., Fresnais J., Gossuin Y., Sandre O. (2012). A Universal Scaling Law to Predict the Efficiency of Magnetic Nanoparticles as MRI T_2_-Contrast, Agents. Adv. Healthc. Mater..

[29] Vuong Q.L., Gossuin Y., Gillis P., Delangre S. (2012). New simulation approach using classical formalism to water nuclear magnetic relaxation dispersions in presence of superparamagnetic particles used as MRI contrast agents. J. Chem. Phys..

[30] Brown M.A., Semalka R.C. (2003). MRI: Basic Principles and Applications.

[31] Estelrich J., Sánchez-Martín M.J., Busquets M.A. (2015). Nanoparticles in magnetic resonance imaging: From simple to dual contrast agents. Int. J. Nanomed..

[32] Lee S.H., Kim B.H., Na H.B., Hyeon T. (2014). Paramagnetic inorganic nanoparticles as T_1_ MRI contrast agents. Wiley Interdiscip. Rev. Nanomed. Nanobiotechnol..

[33] Bridot J.-L., Faure A.-C., Laurent S., Rivière C., Billotey C., Hiba B., Janier M., Josserand V., Coll J.-L., Vander Elst L. (2007). Hybrid gadolinium oxide nanoparticles: Multimodal contrast agents for in vivo imaging. J. Am. Chem. Soc..

[34] Zhang W., Martinelli J., Mayer F., Bonnet C.S., Szeremeta F., Djanashvili K. (2015). Molecular architecture control in synthesis of spherical Ln-containing nanoparticles. RSC Adv..

[35] Osseni S.A., Lechevallier S., Verelst M., Perriat P., Dexpert-Ghys J., Neumeyer D., Garcia R., Mayer F., Djanashvili K., Peters J.A. (2014). Gadolinium oxysulfide nanoparticles as multimodal imaging agents for T_2_-weighted MR, X-ray tomography and photoluminescence. Nanoscale.

[36] Mayer F., Peters J.A., Djanashvili K. (2012). Microwave-assisted seeded growth of lanthanide-based nanoparticles for imaging and therapy. Chem. Eur. J..

[37] Cheung E.N.M., Alvares R.D.A., Oakden W., Chaudhary R., Hill M.L., Pichaandi J., Mo G.C.H., Yip C., Macdonald P.M., Stanisz G.P. (2010). Polymer-Stabilized Lanthanide Fluoride Nanoparticle Aggregates as Contrast Agents for Magnetic Resonance Imaging and Computed Tomography. Chem. Mater..

[38] Evanics F., Diamente P.R., Van Veggel F.C.J.M., Stanisz G.J., Prosser R.S. (2006). Water-soluble GdF_3_ and GdF_3_/LaF_3_ nanoparticles-physical characterization and NMR relaxation properties. Chem. Mater..

[39] Hifumi H., Yamaoka S., Tanimoto A., Citterio D., Suzuki K. (2006). Gadolinium-based hybrid nanoparticles as a positive MR contrast agent. J. Am. Chem. Soc..

[40] Frangville C., Gallois M., Li Y., Nguyen H.H., Lauth-de Viguerie N., Talham D.R., Mingotaud C., Marty J.-D. (2016). Hyperbranched polymer mediated size-controlled synthesis of gadolinium phosphate nanoparticles: Colloidal properties and particle size-dependence on MRI relaxivity. Nanoscale.

[41] Park J.Y., Baek M.J., Choi E.S., Woo S., Kim J.H., Kim T.J., Jung J.C., Chae K.S., Chang Y., Lee G.H. (2009). Paramagnetic ultrasmall gadolinium oxide nanoparticles as advanced T_1_ MRI contrast agent: Account for large longitudinal relaxivity, optimal particle diameter, and in vivo T_1_ MR images. ACS Nano.

[42] Yang J., Shan P., Zhao Q., Zhang S., Li L., Yang X., Yu X., Lu Z., Wang Z., Zhang X. (2021). A design strategy of ultrasmall Gd_2_O_3_ nanoparticles for T_1_ MRI with high performance. New J. Chem..

[43] Dai Y., Wu C., Wang S., Li Q., Zhang M., Li J., Xu K. (2018). Comparative study on in vivo behavior of PEGylated gadolinium oxide nanoparticles and Magnevist as MRI contrast agent. Nanomed. Nanotechnol. Biol. Med..

[44] Park Y., Kim H.M., Kim J.H., Moon K.C., Yoo B., Lee K.T., Lee N., Choi Y., Park W., Ling D. (2012). Theranostic probe based on lanthanide-doped nanoparticles for simultaneous in vivo dual-modal imaging and photodynamic therapy. Adv. Mater..

[45] Pan D., Schmieder A.H., Wickline S.A., Lanza G.M. (2011). Manganese-based MRI contrast agents: Past, present and future. Tetrahedron.

[46] Cai X., Zhu Q., Zeng Y., Zeng Q., Chen X., Zhan Y. (2019). Manganese Oxide Nanoparticles as MRI Contrast Agents in tumour multimodal imaging and therapy. Int. J. Nanomed..

[47] Ding B., Zheng P., Ma P.A., Lin J. (2020). Manganese Oxide Nanomaterials: Synthesis, Properties, and Theranostic Applications. Adv. Mater..

[48] Shin J., Anisur R.M., Ko M.K., Im G.H., Lee J.H., Lee I.S. (2009). Hollow Manganese Oxide Nanoparticles as Multifunctional Agents for Magnetic Resonance Imaging and Drug Delivery. Angew. Chem. Int. Ed..

[49] Wei R., Liu K., Zhang K., Fan Y., Lin H., Gao J. (2022). Zwitterion-Coated Ultrasmall MnO Nanoparticles Enable Highly Sensitive T_1_-Weighted Contrast-Enhanced Brain Imaging. ACS Appl. Mater. Interfaces.

[50] Li J., Wu C., Hou P., Zhang M., Xu K. (2018). One-pot preparation of hydrophilic manganese oxide nanoparticles as T_1_ nano-contrast agent for molecular magnetic resonance imaging of renal carcinoma in vitro and in vivo. Biosens. Bioelectron..

[51] Xiao J., Tian X.M., Yang C., Liu P., Luo N.Q., Liang Y., Li H.B., Chen D.H., Wang C.X., Li L. (2013). Ultrahigh relaxivity and safe probes of manganese oxide nanoparticles for in vivo imaging. Sci. Rep..

[52] Botta M., Geraldes C.F.G.C., Tei L. (2022). High spin Fe(III)-doped nanostructures as T_1_ MR imaging probes. Wiley Interdiscip. Rev. Nanomed. Nanobiotechnol..

[53] Zhao Z., Zhou Z., Bao J., Wang Z., Hu J., Chi X., Ni K., Wang R., Chen X., Chen Z. (2013). Octapod iron oxide nanoparticles as high-performance T_2_ contrast agents for magnetic resonance imaging. Nat. Commun..

[54] Wang Y., Xu C., Chang Y., Zhao L., Zhang K., Zhao Y., Gao F., Gao X. (2017). Ultrasmall Superparamagnetic Iron Oxide Nanoparticle for T_2_-Weighted Magnetic Resonance Imaging. ACS Appl. Mater. Interfaces.

[55] Leal M.P., Muñoz-Hernández C., Berry C.C., García-Martín M.L. (2015). In vivo pharmacokinetics of T_2_ contrast agents based on iron oxide nanoparticles: Optimization of blood circulation times. RSC Adv..

[56] Lee N., Choi Y., Lee Y., Park M., Moon W.K., Choi S.H., Hyeon T. (2012). Water-dispersible ferrimagnetic iron oxide nanocubes with extremely high r2 relaxivity for highly sensitive in vivo MRI of tumors. Nano Lett..

[57] Caro C., Paez-Muñoz J.M., Beltrán A.M., Leal M.P., García-Martín M.L. (2021). PEGylated Terbium-Based Nanorods as Multimodal Bioimaging Contrast Agents. ACS Appl. Nano Mater..

[58] Hu H., Zhang Y., Shukla S., Gu Y., Yu X., Steinmetz N.F. (2017). Dysprosium-Modified Tobacco Mosaic Virus Nanoparticles for Ultra-High-Field Magnetic Resonance and Near-Infrared Fluorescence Imaging of Prostate Cancer. ACS Nano.

[59] Zheng X.-Y., Pellico J., Khrapitchev A.A., Sibson N.R., Davis J.J. (2018). Dy-DOTA integrated mesoporous silica nanoparticles as promising ultrahigh field magnetic resonance imaging contrast agents. Nanoscale.

[60] Ni D., Zhang J., Bu W., Zhang C., Yao Z., Xing H., Wang J., Duan F., Liu Y., Fan W. (2016). PEGylated NaHoF_4_ nanoparticles as contrast agents for both X-ray computed tomography and ultra-high field magnetic resonance imaging. Biomaterials.

[61] Caravan P., Greenfield M.T., Bulte J.W.M. (2001). Molecular factors that determine Curie spin relaxation in dysprosium complexes. Magn. Reson. Med..

[62] Norek M., Kampert E., Zeitler U., Peters J.A. (2008). Tuning of the size of Dy_2_O_3_ nanoparticles for optimal performance as an MRI contrast agent. J. Am. Chem. Soc..

[63] Wang G., Peng Q., Li Y. (2011). Lanthanide-doped nanocrystals: Synthesis, optical-magnetic properties, and applications. Acc. Chem. Res..

[64] Das G.K., Johnson N.J.J., Cramen J., Blasiak B., Latta P., Tomanek B., van Veggel F.C.J.M. (2012). NaDyF_4_ Nanoparticles as T_2_ Contrast Agents for Ultrahigh Field Magnetic Resonance Imaging. J. Phys. Chem. Lett..

[65] Jun Y.-W., Seo J.-W., Cheon J. (2008). Nanoscaling Laws of Magnetic Nanoparticles and Their Applicabilities in Biomedical Sciences. Acc. Chem. Res..

[66] McCarthy J.R., Weissleder R. (2008). Multifunctional magnetic nanoparticles for targeted imaging and therapy. Adv. Drug Deliv. Rev..

[67] Gao J., Gu H., Xu B. (2009). Multifunctional Magnetic Nanoparticles: Design, Synthesis, and Biomedical Applications. Acc. Chem. Res..

[68] Zhang L., Liu R., Peng H., Li P., Xu Z., Whittaker A.K. (2016). The evolution of gadolinium-based contrast agents: From single-modality to multi-modality. Nanoscale.

[69] Yoo D., Lee J.-H., Shin T.-H., Cheon J. (2011). Theranostic Magnetic Nanoparticles. Acc. Chem. Res..

[70] Xu W., Kattel K., Park Y., Chang Y., Kim T., Gang J., Lee H. (2012). Paramagnetic nanoparticle T_1_ and T_2_ MRI contrast agents. Phys. Chem. Chem. Phys..

[71] Zhou Z., Bai R., Munasinghe J., Shen Z., Nie L., Chen X. (2017). T_1_–T_2_ Dual-Modal Magnetic Resonance Imaging: From Molecular Basis to Contrast Agents. ACS Nano.

[72] Tegafaw S.L., Ahmad M.Y., Al Saidi A.K.A., Zhao D., Liu Y., Nam S.-W., Chang Y., Lee G.H. (2023). Magnetic Nanoparticle-Based High-Performance Positive and Negative Magnetic Resonance Imaging Contrast Agents. Pharmaceutics.

[73] Courant T., Roullin V.G., Cadiou C., Callewaert M., Andry M.C., Portefaix C., Hoeffel C., de Goltstein M.C., Port M., Laurent S. (2012). Hydrogels Incorporating GdDOTA: Towards Highly Efficient Dual T_1_/T_2_ MRI Contrast Agents. Angew. Chem. Int. Ed..

[74] Marasini S., Yue H., Ghazanfari A., Ho S.L., Park J.A., Kim S., Cha H., Liu S., Tegafaw T., Ahmad M.Y. (2021). Polyaspartic Acid-Coated Paramagnetic Gadolinium Oxide Nanoparticles as a Dual-Modal T_1_ and T_2_ Magnetic Resonance Imaging Contrast Agent. Appl. Sci..

[75] Mekuria S.L., Debele T.A., Tsai H.-C. (2017). Encapsulation of Gadolinium Oxide Nanoparticle (Gd_2_O_3_) Contrasting Agents in PAMAM Dendrimer Templates for Enhanced Magnetic Resonance Imaging in vivo. ACS Appl. Mater. Interfaces.

[76] Wang L., Lin H., Ma L., Jin J., Shen T., Wei R., Wang X., Ai H., Chen Z., Gao J. (2017). Albumin-Based Nanoparticles Loaded with Hydrophobic Gadolinium Chelates as T_1_−T_2_ Dual-Mode Contrast Agents for Accurate Liver Tumor Imaging. Nanoscale.

[77] Niu D., Luo X., Li Y., Liu X., Wang X., Shi J. (2013). Manganese-loaded dual-mesoporous silica spheres for efficient T_1_- and T_2_-weighted dual mode magnetic resonance imaging. ACS Appl. Mater. Interfaces.

[78] Dong Z., Liang P., Guan G., Yin B., Wang B., Yue R., Zhang X., Song G. (2022). Overcoming Hypoxia-Induced Ferroptosis Resistance via a ^19^F/^1^HMRI Traceable Core-Shell Nanostructure. Angew. Chem. Int. Ed..

[79] Hou X., Yang X., Xu Y., Lin J., Zhang F., Duan X., Liu S., Liu J., Shen J., Shuai X. (2023). Manganese-doped mesoporous polydopamine nanoagent for T_1_–T_2_ magnetic resonance imaging and tumor therapy. Nano Res..

[80] Tegafaw T., Xu W., Ahmad M.W., Baeck J.S., Chang Y., Bae J.E., Chae K.S., Kim T.J., Lee G.H. (2015). Dual-mode T_1_ and T_2_ magnetic resonance imaging contrast agent based on ultrasmall mixed gadolinium-dysprosium oxide nanoparticles: Synthesis, characterization, and in vivo application. Nanotechnology.

[81] Jin X., Fang F., Liu J., Jiang C., Han X., Song Z., Chen J., Sun G., Lei H., Lu L. (2015). An ultrasmall and metabolizable PEGylated NaGdF_4_:Dy nanoprobe for high-performance T_1_/T_2_-weighted MR and CT multimodal imaging. Nanoscale.

[82] Chen Y., Ai K., Liu J., Ren X., Jiang C., Lu L. (2016). Polydopamine-Based Coordination Nanocomplex for T_1_/T_2_ Dual Mode Magnetic Resonance Imaging-Guided Chemo-Photothermal Synergistic Therapy. Biomaterials.

[83] Suárez-García S., Arias-Ramos N., Frias C., Candiota A.P., Arús C., Lorenzo J., Ruiz-Molina D., Novio F. (2018). Dual T_1_/T_2_ Nanoscale Coordination Polymers as Novel Contrast Agents for MRI: A Preclinical Study for Brain Tumor. ACS Appl. Mater. Interfaces.

[84] Chen C., Huang C., Liu J., Tao J., Chen Y., Deng K., Xu Y., Lin B., Zhao P. (2022). Hofmeister Effect-Based T_1_−T_2_ Dual-Mode MRI and Enhanced Synergistic Therapy of Tumor. ACS Appl. Mater. Interfaces.

[85] Li Z., Yi P.W., Sun Q., Lei H., Zhao H.L., Zhu Z.H., Smith S.C., Lan M.B., Lu G.Q. (2012). Ultrasmall Water-Soluble and Biocompatible Magnetic Iron Oxide Nanoparticles as Positive and Negative Dual Contrast Agents. Adv. Funct. Mater..

[86] Wang G., Zhang X., Skallberg A., Liu Y., Hu Z., Mei X., Uvdal K. (2014). One-step synthesis of water-dispersible ultra-small Fe_3_O_4_ nanoparticles as contrast agents for T_1_ and T_2_ magnetic resonance imaging. Nanoscale.

[87] Miao C., Hu F., Rui Y., Duan Y., Gu H. (2019). A T_1_/T_2_ dual functional iron oxide MRI contrast agent with super stability and low hypersensitivity benefited by ultrahigh carboxyl group density. J. Mater. Chem. B.

[88] Wang J., Jia Y., Wang Q., Liang Z., Han G., Wang Z., Lee J., Zhao M., Li F., Bai R. (2021). An Ultrahigh-Field-Tailored T_1_–T_2_ Dual-Mode MRI Contrast Agent for High-Performance Vascular Imaging. Adv Mater..

[89] Zhou Z., Zhao Z., Zhang H., Wang Z., Chen X., Wang R., Chen Z., Gao J. (2014). Interplay between longitudinal and transverse contrasts in Fe_3_O_4_ nanoplates with (111) exposed surfaces. ACS Nano.

[90] Sharma V.K., Alipour A., Soran-Erdem Z., Aykut Z.G., Demir H.V. (2015). Highly monodisperse low-magnetization magnetite nanocubes as simultaneous T_1_–T_2_ MRI contrast agents. Nanoscale.

[91] Thapa B., Diaz-Diestra D., Santiago-Medina C., Kumar N., Tu K., Beltran-Huarac J., Jadwisienczak W.M., Weiner B.R., Morell G. (2018). T_1_- and T_2_-weighted Magnetic Resonance Dual Contrast by Single Core Truncated Cubic Iron Oxide Nanoparticles with Abrupt Cellular Internalization and Immune Evasion. ACS Appl. Bio. Mater..

[92] Li F., Liang Z., Liu J., Sun J., Hu X., Zhao M., Liu J., Bai R., Kim D., Sun X. (2019). Dynamically Reversible Iron Oxide Nanoparticle Assemblies for Targeted Amplification of T_1_-Weighted Magnetic Resonance Imaging of Tumors. Nano Lett..

[93] Li X., Lu S., Xiong Z., Hu Y., Ma D., Lou W., Peng C., Shen M., Shi X. (2019). Light-Addressable Nanoclusters of Ultrasmall Iron Oxide Nanoparticles for Enhanced and Dynamic Magnetic Resonance Imaging of Arthritis. Adv. Sci..

[94] Cao Y., He Y., Mao Z., Kuang Y., Liu M., Zhang Y., Pei R. (2020). Synergistic regulation of longitudinal and transverse relaxivity of extremely small iron oxide nanoparticles (ESIONPs) using pH-responsive nanoassemblies. Nanoscale.

[95] He Y., Cao Y., Mao Z., Zhou Y., Zhang Y., Pei R. (2021). Redox-triggered aggregation of ESIONPs with switchable T_1_ to T_2_ contrast effect for T_2_-weighted magnetic resonance imaging. J. Mater. Chem. B.

[96] He Y., Mao Z., Lu Z., Yan J., Zhang Y., Bianco A., Cao Y., Pei R. (2022). Extremely Small Iron Oxide Nanoparticles with pH-Dependent Solubility Transition as T_1_/T_2_ Switchable Contrast Agents for MRI. ACS Appl. Nano Mater..

[97] He Y., Mao Z., Zhang Y., Lu H., Yan J., Cao Y., Pei R. (2020). Tumor Acid Microenvironment-Triggered Self-Assembly of ESIONPs for T_1_/T_2_ Switchable Magnetic Resonance Imaging. ACS Appl. Bio Mater..

[98] Wang L., Huang J., Chen H., Wu H., Xu Y., Li Y., Yi H., Wang Y.A., Yang L., Mao H. (2017). Exerting Enhanced Permeability and Retention Effect Driven Delivery by Ultrafine Iron Oxide Nanoparticles with T_1_–T_2_ Switchable Magnetic Resonance Imaging Contrast. ACS Nano.

[99] Wang C., Yan C., An L., Zhao H., Song S., Yang S. (2021). Fe_3_O_4_ assembly for tumor accurate diagnosis by endogenous GSH responsive T_2_/T_1_ magnetic relaxation conversion. J. Mater. Chem. B.

[100] Zhou H., Tang J., Li J., Li W., Liu Y., Chen C. (2017). In vivo aggregation-induced transition between T_1_ and T_2_ relaxations of magnetic ultra-small iron oxide nanoparticles in tumor microenvironment. Nanoscale.

[101] Si G., Hapuarachchige S., Artemov D. (2022). Ultrasmall Superparamagnetic Iron Oxide Nanoparticles as Nanocarriers for Magnetic Resonance Imaging: Development and In Vivo Characterization. ACS Appl. Nano Mater..

[102] Choi J.-s., Kim S., Yoo D., Shin T.-H., Kim H., Gomes M.D., Kim S.H., Pines A., Cheon J. (2017). Distance-dependent magnetic resonance tuning as a versatile MRI sensing platform for biological targets. Nat. Mater..

[103] Zhou Z., Huang D., Bao J., Chen Q., Liu G., Chen Z., Chen X., Gao J. (2012). A Synergistically Enhanced T_1_–T_2_ Dual-Modal Contrast Agent. Adv. Mater..

[104] Gupta A.K., Gupta M. (2005). Synthesis and surface engineering of iron oxide nanoparticles for biomedical applications. Biomaterials.

[105] Choi J.-s., Lee J.-H., Shin T.-H., Song H.-T., Kim E.Y., Cheon J. (2010). Self-confirming “AND” logic nanoparticles for fault-free MRI. J. Am. Chem. Soc..

[106] Yang M., Gao L., Liu K., Luo C., Wang Y., Yu L., Peng H., Zhang W. (2015). Characterization of Fe_3_O_4_/SiO_2_/Gd_2_O(CO_3_)_2_ core/shell/shell nanoparticles as T_1_ and T_2_ dual mode MRI contrast agent. Talanta.

[107] Li J., You J., Wu C., Dai Y., Shi M., Dong L., Xu K. (2018). T_1_–T_2_ molecular magnetic resonance imaging of renal carcinoma cells based on nano-contrast agents. Int. J. Nanomed..

[108] Li F., Zhi D., Luo Y., Zhang J., Nan X., Zhang Y., Zhou W., Qiu B., Wen L., Liang G. (2016). Core/shell Fe_3_O_4_/Gd_2_O_3_ nanocubes as T_1_–T_2_ dual modal MRI contrast agents. Nanoscale.

[109] Im G.H., Kim S.M., Lee D.-G., Lee W.J., Lee J.H., Lee I.S. (2013). Fe_3_O_4_/MnO hybrid nanocrystals as a dual contrast agent for both T_1_*-* and T_2_-weighted liver MRI. Biomaterials.

[110] Kim M.H., Son H.-Y., Kim G.-Y., Park K., Huh Y.-M., Haam S. (2016). Redoxable heteronanocrystals functioning magnetic relaxation switch for activatable T_1_ and T_2_ dual-mode magnetic resonance imaging. Biomaterials.

[111] Ren S., Yang J., Ma J., Li X., Wu W., Liu C., He J., Miao L. (2018). Ternary-Responsive Drug Delivery with Activatable Dual Mode Contrast Enhanced in vivo Imaging. ACS Appl. Mater. Interfaces.

[112] Shin T.-H., Choi J.-s., Yun S., Kim I.-S., Song H.-T., Kim Y., Park K.I., Cheon J. (2014). T_1_ and T_2_ dual-mode MRI contrast agent for enhancing accuracy by engineered nanomaterials. ACS Nano.

[113] Keasberry N.A., Bañobre-López M., Wood C., Stasiuk G.J., Gallo J., Long N.J. (2015). Tuning the relaxation rates of dual-mode T_1_/T_2_ nanoparticle contrast agents: A study into the ideal system. Nanoscale.

[114] Bae K.H., Kim Y.B., Lee Y., Hwang J., Park H., Park T.G. (2010). Bioinspired Synthesis and Characterization of Gadolinium-Labeled Magnetite Nanoparticles for Dual Contrast T_1_- and T_2_-Weighted Magnetic Resonance Imaging. Bioconjug. Chem..

[115] Shen J., Li Y., Zhu Y., Yang X., Yao X., Li J., Huang G., Li C. (2015). Multifunctional gadolinium-labeled silica-coated Fe_3_O_4_ and CuInS_2_ nanoparticles as a platform for in vivo tri-modality magnetic resonance and fluorescence imaging. J. Mater. Chem. B.

[116] Wang Z., Liu J., Li T., Liu J., Wang B. (2014). Controlled synthesis of MnFe_2_O_4_ nanoparticles and Gd complex-based nanocomposites as tunable and enhanced T_1_/T_2_-weighted MRI contrast agents. J. Mater. Chem. B.

[117] Yang H., Zhuang Y., Sun Y., Dai A., Shi X., Wu D., Li F., Hu H., Yang S. (2011). Targeted dual contrast T_1_- and T_2_-weighted magnetic resonance imaging of tumors using multifunctional gadolinium-labeled superparamagnetic iron oxide nanoparticles. Biomaterials.

[118] Pinho S.L.C., Sereno J., Abrunhosa A.J., Delville M.H., Rocha J., Carlos L.D., Geraldes C.F.G.C. (2019). Gd- and Eu-Loaded Iron Oxide@Silica Core−Shell Nanocomposites as Trimodal Contrast Agents for Magnetic Resonance Imaging and Optical Imaging. Inorg. Chem..

[119] Bomatí M.O., Gossuin Y., Morales M.P., Gillis P., Muller R.N., Veintemillas-Verdaguer S. (2007). Comparative analysis of the 1H NMR relaxation enhancement produced by iron oxide and core-shell iron–iron oxide nanoparticles. Magn. Reson. Imaging.

[120] Wang K., An L., Tian Q., Lin J., Yang S. (2018). Gadolinium-labelled iron/iron oxide core/shell nanoparticles as T_1_–T_2_ contrast agent for magnetic resonance imaging. RSC Adv..

[121] Li J., Li X., Gong S., Zhang C., Qian C., Qiao H., Sun M. (2020). Dual-Mode Avocado-like All-Iron Nanoplatform for Enhanced T_1_/T_2_ MRI-Guided Cancer Theranostic Therapy. Nano Lett..

[122] Shultz M.D., Calvin S., Fatouros P.P., Morrison S.A., Carpenter E.E. (2007). Enhanced ferrite nanoparticles as MRI contrast agents. J. Magn. Magn. Mater..

[123] Wang X., Zhou Z., Wang Z., Xue Y., Zeng Y., Gao J., Zhu L., Zhang X., Liu G., Chen X. (2013). Gadolinium embedded iron oxide nanoclusters as T_1_–T_2_ dual-modal MRI-visible vectors for safe and efficient siRNA delivery. Nanoscale.

[124] Zhou Z., Wang L., Chi X., Bao J., Yang L., Zhao W., Chen Z., Wang X., Chen X., Gao J. (2013). Engineered Iron-Oxide-Based Nanoparticles as Enhanced T_1_ Contrast Agents for Efficient Tumor Imaging. ACS Nano.

[125] Huang G., Li H., Chen J., Zhao Z., Yang L., Chi X., Chen Z., Wang X., Gao J. (2014). Tunable T_1_ and T_2_ contrast abilities of manganese-engineered iron oxide nanoparticles through size control. Nanoscale.

[126] Yang L., Zhou Z., Liu H., Wu C., Zhang H., Huang G., Ai H., Gao J. (2015). Europium-engineered iron oxide nanocubes with high T_1_ and T_2_ contrast abilities for MRI in living subjects. Nanoscale.

[127] Cheng K., Yang M., Zhang R., Qin C., Su X., Cheng Z. (2014). Hybrid nanotrimers for dual T_1_ and T_2_-weighted magnetic resonance imaging. ACS Nano.

[128] Zhang A., Meng K., Liu Y., Pan Y., Qu W., Chen D., Xie S. (2020). Absorption, distribution, metabolism, and excretion of nanocarriers in vivo and their influences. Adv. Colloid Interface Sci..

[129] Roemhild K., von Maltzahn F., Weiskirchen R., Knüchel R., von Stillfried S., Lammers T. (2021). Iron metabolism: Pathophysiology and Pharmacology. Trends Pharmacol. Sci..

[130] Lucchini R.G., Martin C.J., Doney B.C. (2009). From Manganism to Manganese-Induced Parkinsonism: A Conceptual Model Based on the Evolution of Exposure. Neuromol. Med..

[131] Alromi D.A., Madani S.Y., Seifalian A. (2021). Emerging application of magnetic nanoparticles for diagnosis and treatment of cancer. Polymers.

[132] Choi H.S., Liu W., Misra P., Tanaka E., Zimmer J.P., Ipe B.I., Bawendi M.G., Frangioni J.V. (2007). Renal clearance of nanoparticles. Nat. Biotechnol..

[133] Mou X., Ali Z., Li S., He N. (2015). Applications of Magnetic Nanoparticles in Targeted Drug Delivery System. J. Nanosci. Nanotechnol..

[134] Chen J., Jiang Z., Zhang Y.S., Ding J., Chen X. (2021). Smart transformable nanoparticles for enhanced tumor theranostics. Appl. Phys. Rev..

[135] Baetke S.C., Lammers T., Kiessling F. (2015). Applications of nanoparticles for diagnosis and therapy of cancer. Br. J. Radiol..

[136] Nunn A.D.P. (2006). The cost of developing imaging agents for routine clinical use. Investig. Radiol..

[137] Josephson L., Rudin M. (2013). Barriers to clinical translation with diagnostic drugs. J. Nucl. Med..

